# Advice to use infant formula and free samples are common in both urban and rural areas in China: a cross-sectional survey

**DOI:** 10.1017/S1368980020005364

**Published:** 2021-06

**Authors:** Jia Li, Tuan T Nguyen, Yifan Duan, Roger Mathisen, Zhenyu Yang

**Affiliations:** 1 School of Business, Nanjing University of Information Science & Technology, Nanjing, People’s Republic of China; 2 Alive & Thrive Southeast Asia, FHI 360, Hanoi, Vietnam; 3 National Institute for Nutrition and Health, Chinese Center for Disease Control and Prevention, No.27 Nanwei Road, Xicheng District, Beijing 100050, People’s Republic of China

**Keywords:** Breast-feeding support, China, Cross-promotion, Infant formula, International Code of Marketing of Breastmilk Substitutes

## Abstract

**Objective:**

To examine the association between the place of residence and receiving free samples and advice to feed the baby with infant formula.

**Design:**

A cross-sectional study.

**Setting:**

The current study covered twelve counties/districts in China.

**Participants:**

5112 mothers with infants aged 0–5·9 months.

**Results:**

About 16 % of the mothers received free samples of infant formula. During pregnancy, this likelihood was higher among mothers in small and medium cities (OR: 1·96; 95 % CI 1·14, 3·38) and non-poor rural counties (OR: 4·65; 95 % CI 1·65, 13·14) compared with mothers in big cities. During the hospital stay, it was lower in big cities. After discharge, it was lower in poor rural counties (OR: 0·14; 95 % CI 0·05, 0·41). About 26 % of the mothers were advised to feed their infants with infant formula. The likelihood of receiving advice to feed the baby with infant formula from hospitals was lower in non-poor (OR: 0·37; 95 % CI 0·21, 0·66) and poor rural counties (OR: 0·35; 95 % CI 0·13, 0·91) than in big cities. Mothers in non-poor rural counties were less likely to receive advice from traditional mass media (OR: 0·17; 95 % CI 0·06, 0·48), while mothers in small and medium cities were more likely to receive advice from modern mass media (OR: 1·84; 95 % CI 1·20, 2·80) compared with mothers in big cities.

**Conclusions:**

The promotion strategy of infant formula varies from different places of residence in China. The study suggests the need to strengthen enforcement of relevant regulations, especially within health facilities and through modern mass media.

Aggressive promotion of breastmilk substitutes (BMS) is one of the key barriers to successful breast-feeding and thus poses dangers to infant health^(1,2)^. Previous studies showed that inappropriate marketing of BMS affects breast-feeding behaviours of women as well as medical practice of healthcare workers related to supporting breast-feeding^(3–5)^. For example, misconceptions related to feeding and intention to feed infant formula at birth were associated with increased feeding of infant formula in the first 3 d of life, which is associated with increased feeding of infant formula and premature cessation of breast-feeding^(6)^. Studies in China also revealed that mothers who received advice to feed the baby with BMS had a lower prevalence of exclusive breast-feeding and continued breast-feeding^(7,8)^. Promotion of infant formula hinders progress towards achieving the Global Nutrition Targets and Sustainable Development Goals endorsed by most governments^(9)^.

Infant formula sales are high and increasing in East Asia, including in China^(3,10,11)^. Indeed, China is the largest infant formula market in the world^(3)^. The sale of baby food in China, around 90 % of which was formula milk, doubled in just 5 years from 2010 to 2014, and then one and a half fold from 2014 to 2018^(12,13)^. BMS manufacturers and distributors employ various tactics to promote their products. In addition to direct promotion to mothers and their families via television, social media and home visits, manufacturers and distributors promote BMS indirectly via incentives, free samples and activities within the health service setting during pregnancy, hospital stay and after discharge^(1,4,14–17)^. Distribution of free samples of infant formula or promotional materials to pregnant women typically happens during consultations and events organised by BMS manufacturers and distributors, where they familiarise women with the products and aim to increase the preference for certain types of infant formula to use in the future^(18,19)^. The promotion of infant formula during the hospital stay and after discharge through health workers and BMS representatives is also common in China^(20,21)^. Additionally, cross-promotion (e.g., similar labelling, ambiguous messages) across BMS product categories (e.g., milk formula for pregnant women, preterm babies, infants and toddlers) is a common practice of BMS manufacturers and distributors to circumvent the national Code legislation, creating confusion for families^(2)^.

Given the negative impact of BMS promotion and aggressive marketing tactics, regulating the inappropriate practices of BMS companies has been identified as a critical intervention to protect and build supportive environments for breast-feeding^(22)^. The World Health Assembly adopted the *International Code of Marketing of Breastmilk Substitutes* (hereafter *the Code*) in 1981 to prohibit the advertising and promotion of BMS^(23)^. Even though 136 out of 194 countries have adopted some forms of national legal measures adhering to *the Code* by 2020, robust measures are in place in only a few countries to eliminate inappropriate promotion of BMS^(24)^. According to this report, only some provisions of *the Code* are included in the current regulations in China^(24)^. The main policy, *Administrative Measures for the Marketing of Breastmilk Substitutes* (hereafter *the Measure*) issued in 1995^(25)^, was abolished in 2017, leaving the marketing of BMS in China weakly regulated (online supplementary Appendix A).

Furthermore, the China Food and Drug Administration introduced a new regulation in 2016 to establish standards for infant formula^(26,27)^, creating opportunities for certain brands to market themselves as high quality to help expand their market into medium and small cities and rural areas where substandard products were commonly used^(28)^. As the Chinese economy develops, rising income makes infant formula more affordable for mothers in lower socio-economic groups (e.g., rural or urban poor). Introduction of the universal two-child policy in 2015 is projected to result in a significant increase in birth rate, especially in small cities and rural areas of China^(29)^. BMS companies might have captured those policy gaps and socio-demographic dynamic changes to expand their market in China. Previous studies in China have focused on the promotion of infant formula in big cities^(7,20)^. Thus, information about the promotion of infant formula in less-urbanised areas in China is limited.

To address this gap in the literature, we analysed data from a large-scale population survey to examine the association between the place of residence and receiving free samples and advice to feed the baby with infant formula among mothers with infants aged 0–5·9 months. We hypothesised that the promotion of BMS varied across places of residence.

## Methods

### Study design and data collection

In the current study, we used secondary data from 10 408 mothers with infants aged 0–11 months old who participated in a cross-sectional survey on determinants of breast-feeding practices in China^(8)^. Infants were approximately equally distributed across each month group. A more detailed description of the study has been described elsewhere^(8)^. To summarise, survey samples were selected via a multi-stage stratified cluster sampling approach. All districts/counties were categorised into four strata, namely big cities, small and medium cities, non-poor rural counties and poor rural counties. Among twelve districts/counties selected in the first stage, four were from big cities, four were from small and medium cities, two were from non-poor rural counties and two were from poor rural counties. In the second stage, four clusters were randomly selected via probability proportional to size sampling method. In each selected cluster, the data collection team visited the corresponding immunisation clinic and invited mothers who brought their 0–11-month-old children to the clinic for immunisation to participate in the study. In total, the survey included face-to-face interviews of 10 408 mothers with infants aged 0–11 months old.

Data were collected between September 2017 and January 2018 in collaboration with research teams at the provincial level Center for Disease Control and Prevention. A structured questionnaire was programmed into smartphones or tablets and then used for data collection (online supplementary Appendix B). Written consent from all participants was obtained.

### Study variables

#### Outcome variables

Promotion of infant formula in the current study was defined as having received free samples of infant formula or advice to feed the baby with infant formula, which was assessed using two retrospective questions. The first question was ‘when did the mothers receive free samples of infant formula?’ We examined three time points: (1) during pregnancy, (2) during hospital stay and (3) after discharge as well as an overall measure of receiving free samples of infant formula (No, when the mother did not receive any free samples of infant formula in any of the three time points; and Yes, for any alternative scenarios). The second question was whether the mothers received advice to feed the baby with infant formula, and if yes, from where. We examined four circumstances: (1) from hospitals where the mother gave birth, (2) from traditional mass media (e.g., TV, radio, magazine or book), (3) from modern mass media (e.g., websites, online shopping malls, websites and platform from hospitals or doctors, and social media such as Weibo and WeChat) and (4) from family members, relatives or friends.

#### Exposure variables

The place of residence was the main exposure in the current study. The whole of China can be divided into twenty-three provinces, five autonomous regions, four centrally administered municipalities and two special administrative regions, which are then subdivided into prefectures, counties/districts and townships^(30,31)^. We sampled districts/counties from big cities, small and medium cities, non-poor rural counties and poor rural counties. Big cities refer to central districts of municipalities directly under the Central Government, cities under separate state planning or provincial cities with a population over a million. Small and medium cities refer to all districts/counties except the central districts of big cities and county-level cities. Poor rural counties refer to counties that are key targets in the national poverty alleviation and development programme^(32)^. Non-poor rural counties refer to all remaining counties.

#### Covariate variables

Maternal and paternal characteristics include level of education (primary or below, junior high school, high school or college and university or higher) and occupation (e.g., unemployed, agriculture related, industry related and white-collar or professionals). Maternal age (≤ 25, 26–35 and ≥ 36 years) and ethnicity (Han and others) were also collected. Child and perinatal characteristics consist of age in months, gender, first birth, having at least one antenatal visit, place of birth (maternity facilities at national or provincial levels, at municipal level, at county level and others), caesarean birth, length of stay in health facility after birth if extended (vaginal births: ≥ 4 d; caesarean births: ≥ 7 d) and special care service for the mothers such as hiring a nanny or staying in a postpartum care centre during the first month afterbirth^(33)^. For the first set of outcome variables on receiving free samples of infant formula, we control for all of the above-mentioned covariates. For the second set of outcome variables about receiving advice to feed the baby with infant formula, we also consider additional covariates including reasons for being advised on feeding infant formula (perceived breast-feeding difficulties in hospital and after discharge) and hospital lack of referral for breast-feeding supporting organisations at discharge.

### Statistical analysis

From 10 408 mothers interviewed, we selected 5261 mothers with infants aged 0–5·9 months. Then, we further excluded 149 records (< 3 % of 5261 records) due to missing values in outcome variables and covariates to come up with the final sample for statistical analysis of 5112. Sensitivity analysis with the complete sample displayed very similar findings with those reported in the current study. The selection of mothers with infants aged 0–5·9 months was to minimise recall bias of the exposure to infant formula free sample and advice.

All the data analysis was conducted using survey command in Stata 15.0 (Stata Inc.) to consider survey design effect. First, we used descriptive analyses to report the prevalence of exposure and covariate variables, and stratified outcome variables by the place of residence. Then, we used multivariate logistic regression models to examine factors associated with receiving free samples or receiving advice to feed the baby with infant formula. We used big cities, mothers with a university education or higher, and mothers who worked as white-collar workers or professionals as the reference groups in the multivariate logistic regression models.

## Results

### General characteristics

Among 5112 mothers with infants 0–5 months old (mean age of 2·5 months), 86·1 % belonged to the Han ethnicity, 63·3 % were between 26 and 35 years old, 21·4 % had at least a college degree and 44·7 % participated in business, professional or industry-related occupations (Table 1). Mothers in rural areas tended to be younger, had less education and were less likely to work as white-collar workers or professionals (Table 1). Fathers of infants had similar educational and occupational patterns as mothers (Table 1).

**Table 1:** Characteristics of participants (in percentage)

	All sample (*n* 5112)	Big cities (*n* 1797)	Medium and small cities (*n* 1663)	Non-poor rural areas (*n* 833)	Rural poor areas (*n* 819)
Parental characteristics					
Maternal ethnicity					
Han	86·1	84·1	80·3	99·0	88·9
Maternal age					
≤ 25 years	25·2	13·0	30·4	29·1	37·7
26–35 years	63·3	69·7	60·4	65·3	53·4
≥36 years	11·4	17·3	9·3	5·6	8·9
Maternal education levels					
University or higher	21·4	44·1	15·2	3·1	3·1
High school or college	36·5	39·0	40·2	40·9	19·3
Junior high school	34·9	15·1	34·3	54·0	60·1
Primary or below	7·1	1·8	10·3	1·9	17·6
Maternal occupations					
White-collar or professionals	36·0	57·1	33·0	21·5	10·4
Industry related	8·7	9·6	9·8	8·8	4·2
Agriculture related	17·3	2·8	15·0	16·6	54·3
Unemployed related	38·1	30·5	42·2	53·2	31·1
Paternal education levels					
University or higher	22·1	46·4	14·9	2·8	2·9
High school or college	35·3	38·1	38·1	35·8	23·2
Junior high school	35·8	13·8	37·8	57·4	58·1
Primary or below	6·8	1·7	9·3	4·1	15·8
Paternal occupations					
White-collar or professionals	48·2	70·0	47·1	35·3	15·9
Industry related	28·4	23·3	28·9	45·6	21·0
Agriculture related	18·5	2·9	18·2	17·4	54·6
Unemployed related	4·9	3·8	5·8	1·7	8·5
Child and perinatal characteristics					
The first birth	46·8	52·9	47·8	32·5	45·9
The child was male	50·2	50·4	50·5	50·1	49·2
Had antenatal visits	95·2	93·4	97·4	98·1	91·5
Health facilities the mothers gave births					
National and provincial levels	18·5	44·8	4·0	0·4	8·7
Municipal level	29·3	40·5	38·2	10·2	6·1
County level	50·3	10·1	57·5	88·2	85·2
Others (mostly private)	1·9	4·7	0·3	1·2	0·0
Caesarean delivery	38·8	33·9	43·2	48·0	30·9
Long stay in health facility after births*	32·5	27·8	29·0	15·6	67·0
Had breastfeeding difficulties in hospital	26·3	36·4	21·9	20·8	18·7
Had breast-feeding difficulties after discharge	26·5	39·8	20·0	20·0	17·3
Hired a nanny or stayed in a postpartum care centre during the first month afterbirth	7·6	16·6	4·9	1·1	0·1
Hospital where the woman gave birth did not recommended breast-feeding supporting centre at discharge	92·4	88·2	96·1	98·7	87·9

* Long stay in health facility after births: vaginal births: ≥ 4 d; caesarean births: ≥ 7 d.

About half of the sample were boys and 46·8 % were the first child. Almost all mothers had antenatal care, delivered at a health facility and spent some days after births at the health facility. The prevalence of caesarean births was 38·8 % and 32·5 % of the mothers had an extended stay in a maternity facility after delivery (vaginal births: ≥ 4 d; caesarean births: ≥ 7 d).

About a quarter of mothers felt they had difficulty with breast-feeding during the hospital stay or after discharge. Less than 10 % of the mothers hired a nanny or stayed in a postpartum care centre during the first month after birth, and the majority of whom lived in big cities. Only one of every ten mothers was referred to a breast-feeding supporting centre at discharge.

### Receiving free samples of infant formula

**Fig. 1:**
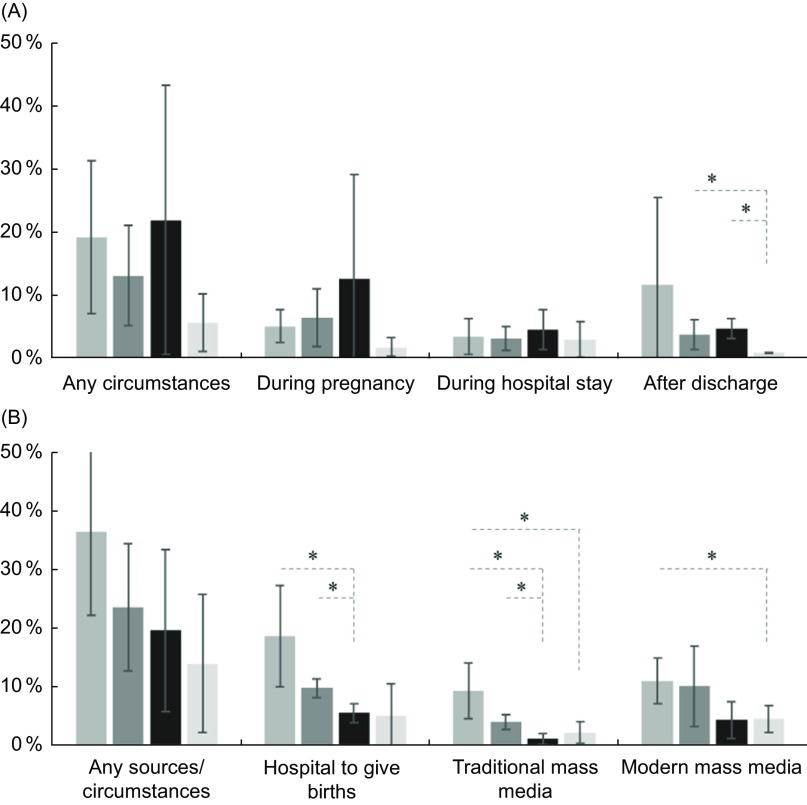
Prevalence (%, 95 % CI) of receiving free samples of infant formula (A) and advice (B) among mothers with infants < 6 months old in China by the place of residence * for non-overlapping 95 % CI. Traditional mass media: TV, radio, magazine or book; modern mass media: Websites, online shopping malls, websites and platform from hospitals or doctors, and social media such as Weibo and WeChat. 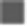, big cities; 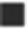, small and medium cities; 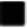, non-poor rural areas; 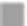, poor rural areas

About 16 % of mothers received free samples of infant formula: 6·3 % received the sample during pregnancy, 3·5 % during their hospital stay and 6·3 % after discharge (Fig. 1). Although there were some variations by the place of residence, the 95 % CI were overlapping in most pairwise comparisons, except for free infant formula sample after discharge: 1 % of mothers in rural poor counties *v*. 4 % in small and medium cities and 5 % in non-poor rural counties (Fig. 1).

In multivariate logistic regression models, the likelihood of receiving free samples of infant formula during pregnancy was higher for mothers in medium and small cities (OR: 1·96; 95 % CI 1·14, 3·38) and in non-poor rural counties (OR: 4·65; 95 % CI 1·65, 13·14) than in big cities (Table 2). This result indicated that mothers in non-poor rural counties were more than four times more likely to receive free samples than those in large cities. Compared with mothers in big cities, mothers in other areas were more likely to receive free samples of infant formula during hospital stay; however, this difference was only significant in non-poor rural counties (OR: 2·06; 95 % CI 1·04, 4·09). In contrast, mothers in big cities were more likely to receive free samples after discharge, especially when compared with mothers in poor rural counties (OR: 0·14; 95 % CI 0·05–0·41) (Table 2). The likelihood of receiving free samples after discharge was higher in mothers working in agricultural sector (OR: 1·99; 95 % CI 1·12, 3·54) or unemployed (OR: 1·59; 95 % CI 1·24, 2·05) than white-collar workers or professionals. The likelihood of mothers who received free samples of infant formula after discharge from private hospitals was higher than received by mothers who gave birth in maternity facilities at national and provincial levels (OR: 1·74; 95 % CI 1·07, 2·84), but lower than received by mothers in maternity facilities at the county (OR: 0·41; 95 % CI 0·26, 0·63) and municipal (OR: 0·51; 95 % CI 0·29, 0·89) levels.

**Table 2:** Factors associated with receiving free samples of infant formula among mothers with infants < 6 months old in China†,‡

	Any circumstances		During pregnancy		During hospital stay		After discharge	
	(*n* 5112)		(*n* 5112)		(*n* 5112)		(*n* 5112)	
	OR	95 % CI	OR	95 % CI	OR	95 % CI	OR	95 % CI
Residence (reference: big cities)								
Small and medium cities	0·94	0·52, 1·70	1·96*	1·14, 3·38	1·44	0·72, 2·88	0·41	0·15, 1·12
Non-poor rural areas	1·76	0·64, 4·82	4·65**	1·65, 13·14	2·06*	1·04, 4·09	0·45	0·17, 1·19
Poor rural areas	0·56	0·19, 1·70	0·98	0·30, 3·19	1·55	0·63, 3·80	0·14***	0·05, 0·41
Parental characteristics								
Maternal age (reference: ≤ 25 years)								
26–35 years	0·97	0·81, 1·17	1·04	0·82, 1·31	0·88	0·65, 1·18	1·03	0·73, 1·44
36+ years	0·72	0·50, 1·02	0·77	0·41, 1·44	0·77	0·45, 1·33	0·77	0·49, 1·21
Maternal ethnicity (reference: others)								
Han	1·96*	1·07, 3·58	1·31	0·67, 2·56	3·18*	1·22, 8·27	2·24*	1·05, 4·75
Maternal education levels (reference: university or higher)								
High school or college	1·01	0·77, 1·30	0·97	0·72, 1·31	0·92	0·54, 1·56	1·05	0·73, 1·51
Junior high school	1·13	0·74, 1·72	1·16	0·95, 1·41	1·26	0·59, 2·69	0·88	0·44, 1·77
Primary or below	0·72	0·35, 1·48	0·34*	0·13, 0·86	0·95	0·29, 3·10	1·09	0·34, 3·51
Maternal occupations (reference: white-collar or professionals)								
Industry related	0·91	0·71, 1·18	1·04	0·74, 1·46	0·67	0·44, 1·02	0·90	0·60, 1·36
Agriculture related	0·74	0·44, 1·26	0·43*	0·22, 0·83	0·54	0·19, 1·51	1·99*	1·12, 3·54
Unemployed	1·01	0·73, 1·40	0·88	0·62, 1·23	0·62	0·33, 1·14	1·59***	1·24, 2·05
Paternal education levels (reference: university or higher)								
High school or college	1·23	0·94, 1·62	0·97	0·66, 1·41	1·39	0·76, 2·51	1·43*	1·02, 2·00
Junior high school	0·98	0·77, 1·24	0·85	0·55, 1·32	0·84	0·35, 2·03	1·32	0·82, 2·12
Primary or below	0·92	0·54, 1·58	1·08	0·52, 2·27	0·35*	0·12, 1·00	1·38	0·48, 3·96
Paternal occupations (reference: white-collar or professionals)								
Industry related	0·91	0·68, 1·22	0·94	0·67, 1·33	0·74	0·42, 1·30	0·94	0·61, 1·43
Agriculture related	0·67**	0·51, 0·87	0·72	0·46, 1·15	1·26	0·79, 2·01	0·21***	0·13, 0·35
Unemployed	0·65	0·42, 1·00	0·58*	0·35, 0·96	0·28	0·06, 1·24	0·83	0·36, 1·93
Child characteristics								
The first birth	1·01	0·85, 1·19	0·91	0·71, 1·16	0·99	0·68, 1·45	1·18	0·88, 1·59
The child was male§	1·06	0·93, 1·20			1·00	0·71, 1·42	1·16	0·96, 1·40
Age in months||	1·07**	1·02, 1·13					1·10*	1·00, 1·21
Perinatal characteristics								
Had antenatal visits	0·99	0·71, 1·39	1·91	0·87, 4·20	0·44**	0·26, 0·73	1·11	0·55, 2·25
Health facilities the mothers gave births (reference: at national or provincial levels)								
Municipal level	0·73	0·47, 1·14	0·95	0·57, 1·56	0·78	0·33, 1·84	0·51*	0·29, 0·89
County level	0·49***	0·34, 0·72	0·55	0·28, 1·06	0·65	0·31, 1·35	0·41***	0·26, 0·63
Others (mostly private facilities)	2·20**	1·22, 3·97	1·64	0·64, 4·22	2·72	0·85, 8·75	1·74*	1·07, 2·84
Caesarean births¶	1·22	1·00, 1·49			1·18	0·94, 1·49	0·99	0·75, 1·30
Long stay in health facility after births††	0·90	0·61, 1·32			0·92	0·69, 1·24	0·64	0·32, 1·31
Hired a nanny or stayed in a postpartum care centre during the first month afterbirth‡‡	0·88	0·41, 1·92					0·40	0·09, 1·72

† Values are adjusted OR and 95 % CI from survey multivariate logistic regression models.

‡ Significantly different from the null value (OR of 1): *P < 0·05, **P < 0·01, ***P < 0·001.

§ Gender of the child was not controlled for since it was unknown during pregnancy.

|| Age in months was not controlled for since it is not related to receiving free samples of infant formula during pregnancy and hospital stay.

¶ Caesarean births were not controlled for since it was unknown during pregnancy.

†† Long stay in health facility after births: vaginal births: ≥ 4 d; caesarean births: ≥ 7 d. It was not controlled for since it was unknown during pregnancy.

‡‡ Hired a nanny or stayed in a postpartum care centre during the first month afterbirth was not controlled for since it happened after discharge.

### Receiving advice to feed the baby with infant formula

About 26 % of mothers in this sample received advice to feed the baby with infant formula: 11·4 % from hospitals, 5·1 % from traditional mass media like TV, radio, magazines and books, 8·6 % from modern mass media such as the internet and social media and 3·1 % from family members, relatives or friends (Fig. 1). More mothers in big cities received this advice from the hospitals and traditional mass media (e.g., TV, radio, magazine and books) than those living in other areas (Fig. 1). Receiving the advice from modern mass media such as internet and social media was higher in big cities than in rural poor counties (non-overlapping 95 % CI) (Fig. 1).

Multivariate logistic regression models showed that the likelihood of receiving advice to feed the baby with infant formula from hospitals was lower in non-poor rural counties (OR: 0·37; 95 % CI 0·21, 0·66) and poor rural counties (OR: 0·35; 95 % CI 0·13, 0·91) than in big cities (Table 3). The likelihood of receiving advice to feed the baby with infant formula from mass media was lower in non-poor rural counties for traditional mass media (OR: 0·17; 95 % CI 0·06, 0·48) but higher in small and medium cities for modern mass media (OR: 1·84; 95 % CI 1·20, 2·80) compared with mothers in big cities. The likelihood of receiving advice to feed the baby with infant formula was similar across most education levels, while it slightly differed across occupations of mothers and fathers (Table 3). Notably, mothers working in industry were more likely to receive advice to feed the baby with infant formula from modern mass media (OR: 1·53; 95 % CI 1·23, 1·91) and family members, relatives or friends (OR: 1·66; 95 % CI1·02, 2·72), and fathers working in industry (OR: 1·44; 95 % CI 1·10, 1·89) for traditional mass media compared with those working as white-collar workers or professionals. The prevalence of receiving advice to feed the baby with infant formula was higher in mothers giving birth in maternity facilities at national and provincial levels than in other level facilities (Table 3). Mothers who encountered breast-feeding difficulties during their hospital stay were more likely to receive advice to feed the baby with infant formula from hospitals (OR: 1·42; 95 % CI 1·05, 1·92), modern mass media (OR: 1·54; 95 % CI 1·21, 1·97) and family members, relatives or friends (OR: 1·60; 95 % CI 1·23, 2·09) (Table 3). Moreover, mothers were more than two times as likely to receive advice of using infant formula from their family members, relatives or friends if they encountered breast-feeding difficulties after discharge (OR: 2·37; 95 % CI 1·69, 3·33).

**Table 3:** Associate factors of receiving advice to feed the baby with infant formula among mothers with infants < 6 months old in China†,‡

	Any sources/circumstances (n 5112)		Hospitals (*n* 5112)		Traditional mass media§ (*n* 5112)		Modern mass media|| (*n* 5112)		Family members, relatives or friend (*n* 5112)	
	OR	95 % CI	OR	95 % CI	OR	95 % CI	OR	95 % CI	OR	95 % CI
Residence (reference: big cities)										
Small and medium cities	0·83	0·45, 1·53	0·65	0·38, 1·11	0·57	0·27, 1·21	1·84**	1·20, 2·80	0·94	0·29–3·00
Non-poor rural areas	0·69	0·34, 1·39	0·37***	0·21, 0·66	0·17***	0·06, 0·48	0·84	0·44, 1·62	1·31	0·52–3·31
Poor rural areas	0·73	0·39, 1·36	0·35*	0·13, 0·91	0·51	0·20, 1·28	1·19	0·73, 1·94	1·80	0·67–4·84
Parental characteristics										
Maternal age (reference: ≤ 25 years)										
26–35 years	1·14	0·93, 1·40	1·11	0·80, 1·54	1·33	0·87, 2·02	1·12	0·80, 1·56	1·10	0·73–1·64
36+ years	1·25	0·93, 1·67	1·18	0·75, 1·87	2·13***	1·36, 3·34	1·20	0·77, 1·87	0·65	0·34–1·26
Maternal ethnicity (reference: others)										
Han	1·30	0·93, 1·81	1·51	0·92, 2·49	1·13	0·61, 2·09	1·61**	1·15, 2·27	0·62	0·32–1·23
Maternal education levels (reference: university or higher)										
High school or college	1·08	0·91, 1·28	1·15	0·80, 1·66	1·38	0·80, 2·38	1·02	0·68, 1·53	0·89	0·60–1·30
Junior high school	0·90	0·71, 1·15	1·11	0·79, 1·57	1·12	0·51, 2·43	0·73	0·43, 1·23	0·70	0·27–1·80
Primary or below	0·65*	0·44, 0·96	1·23	0·75, 2·00	0·72	0·24, 2·18	0·49	0·23, 1·07	0·65	0·20–·16
Maternal occupations (reference: white-collar or professionals)										
Industry related	1·14	0·82, 1·58	0·90	0·50, 1·63	0·94	0·43, 2·06	1·53***	1·23, 1·91	1·66*	1·02–2·72
Agriculture related	0·74	0·46, 1·21	0·93	0·46, 1·86	0·63	0·23, 1·71	1·17	0·67, 2·03	0·50	0·13–1·94
Unemployed	0·99	0·81, 1·21	0·97	0·75, 1·27	0·88	0·66, 1·17	1·19	0·91, 1·57	1·21	0·77–1·90
Paternal education levels (reference: university or higher)										
High school or college	1·26	0·90, 1·77	1·29	0·89, 1·88	0·90	0·66, 1·22	1·08	0·72, 1·61	1·09	0·46–2·57
Junior high school	1·10	0·71, 1·72	1·11	0·69, 1·78	0·84	0·41, 1·71	1·09	0·68, 1·73	0·86	0·25–2·89
Primary or below	1·34	0·75, 2·39	1·47	0·68, 3·17	0·55	0·19, 1·61	1·05	0·54, 2·05	0·40	0·06–2·91
Paternal occupations (reference: white-collar or professionals)										
Industry related	0·86	0·72, 1·02	0·86	0·68, 1·08	1·44**	1·10, 1·89	0·87	0·64, 1·19	0·86	0·58–1·27
Agriculture related	0·59**	0·43, 0·81	0·80	0·47, 1·37	0·77	0·28, 2·13	0·46***	0·31, 0·71	0·41	0·10–1·72
Unemployed	0·74	0·47, 1·15	0·78	0·36, 1·67	0·69	0·32, 1·49	0·43*	0·22, 0·84	1·65	0·63–4·31
Child characteristics										
The first birth	1·20	0·96, 1·51	1·13	0·91, 1·39	1·30	0·89, 1·88	1·17	0·93, 1·47	1·24	0·71–2·15
The child was male	1·03	0·90, 1·18	1·11	0·88, 1·38	1·01	0·78, 1·29	0·93	0·79, 1·08	1·04	0·78–1·39
Age in months	1·10**	1·03, 1·18	1·00	0·92, 1·08	1·09	0·99, 1·19	1·12**	1·04, 1·19	1·21***	1·10–1·33
Perinatal characteristics										
Had antenatal visits	1·20	0·86, 1·68	0·77	0·46, 1·28	1·00	0·44, 2·26	1·15	0·87, 1·52	2·33	0·75–7·23
Health facilities the mothers gave births (reference: at national or provincial levels)										
Municipal level	0·76*	0·61, 0·96	0·66***	0·53, 0·82	1·02	0·59, 1·76	0·58**	0·38, 0·86	1·77*	1·10–2·85
County level	0·64**	0·47, 0·86	0·58***	0·43, 0·78	0·69	0·44, 1·08	0·37***	0·24, 0·55	2·26*	1·03–4·92
Others (mostly private facilities)	1·11	0·50, 2·47	0·63	0·30, 1·31	0·34***	0·24, 0·47	0·32***	0·17, 0·60	0·47	0·12–1·87
Caesarean births	1·21	0·97, 1·52	1·15	0·86, 1·54	1·06	0·84, 1·35	0·98	0·68, 1·41	1·22	0·92–1·64
Long stay in health facility after births††	0·83	0·67, 1·01	1·03	0·86, 1·22	1·09	0·83, 1·42	0·96	0·70, 1·31	0·74	0·47–1·16
Hired a nanny or stayed in a postpartum care centre during the first month afterbirth	1·13	0·71, 1·80	1·11	0·83, 1·49	1·33	0·91, 1·94	1·00	0·71, 1·41		0·40–2·12
Breastfeeding difficulties in hospital	1·53***	1·24, 1·89	1·42*	1·05, 1·92	0·96	0·65, 1·39	1·54***	1·21, 1·97	1·60***	1·23–2·09
Breast-feeding difficulties after discharge	1·36**	1·10, 1·69	1·19	0·93, 1·53	0·91	0·70, 1·20	1·14	0·85, 1·52	2·37***	1·69–3·33
Hospital where the woman gave birth did not recommended breast-feeding support centre at discharge	1·01	0·75, 1·35	1·05	0·78, 1·43	0·75	0·42, 1·34	0·85	0·65, 1·10	0·76	0·39–1·50

† Values are adjusted OR and 95 % CI from survey multivariate logistic regression.

‡ Significantly different from the null value (OR of 1): *P < 0·05, **P < 0·01, ***P < 0·001.

§ Traditional mass media: TV, radio, magazine or book.

|| Modern mass media: Websites, online shopping malls, websites and platform from hospitals or doctors, and social media such as Weibo and WeChat.

†† Long stay in health facility after births: vaginal births: ≥ 4 d; caesarean births: ≥ 7 d.

## Discussions

### Principal results

In the current study, we found that the promotion of infant formula in the form of free samples and advice was common in China. Mothers reported receiving infant formula promotion from multiple channels and at different time points including during pregnancy, hospital stay and after discharge.

First, the likelihood of receiving free samples of infant formula was still high (about 20 % in big cities and 16 % overall). This likelihood is lower than what has been found in previous studies in six big cities in China^(20)^. In a sample of almost 300 mothers recruited from outpatient clinics in seventeen hospitals in 2012, previous study documented that 40 % of the mothers received free samples of infant formula; 76 % received samples in or near hospitals, mostly by BMS sale representatives (61 %) and health workers (37 %)^(20)^. Our study was a population-based study across the country with a sample size of more than 5000 mothers, and thus the findings might not be readily comparable to the hospital-based studies in big cities. The lower prevalence of receiving free samples of infant formula, however, might indicate a certain progress towards promoting breast-feeding in China, including strengthening the baby-friendly environment through baby-friendly hospital reassessment^(34)^ and the implementation of early essential newborn care (EENC)^(35,36)^. Most of the hospitals participating in the studies^(20)^ had been accredited as baby-friendly hospitals and thus would have good background to shift back to a supportive environment for breast-feeding. A recent report by China Consumers Association on the promotion and sales of BMS revealed that mothers who gave birth to their babies in non-baby-friendly hospitals were more likely to use BMS^(37)^. In addition, introduction of EENC in the last decade in China may have helped to improve early and exclusive breast-feeding practices in the hospitals^(35)^, leading to a decline in the use of infant formula and support continuing breast-feeding^(38)^.

In addition, the infant formula promotion in terms of providing free samples in China might have expanded to target mothers in lower socio-economic groups and in rural areas. For example, our data showed that more mothers in small and medium cities and non-poor rural counties received free samples compared with mothers in big cities during pregnancy and their hospital stay. The proportion of mothers who received free samples after hospital discharge was about the same across all areas. Socio-economic status of the mothers and fathers was not a factor, suggesting that BMS manufacturers and distributors are expanding their focus to the whole population. More importantly, the likelihood of mothers receiving free samples of infant formula after discharge was higher in mothers working in agriculture or unemployed than those working as white-collar or professionals. Compared with mothers with university or higher education level, the likelihood of receiving free samples of infant formula after discharge was higher in mothers with high school or college education level. By introducing free samples of infant formula in various locations and time points, the BMS manufacturers and distributors might increase the use and dependence on infant formula^(39)^.

Second, we found that one of every four mothers received advice to feed the baby with infant formula. This prevalence was higher when they gave birth in national or provincial hospitals. In China, patients tended to pursue higher quality healthcare services, including births in tertiary and secondary hospitals^(40)^. To reach more mothers, BMS manufacturers and distributors would target national and provincial hospitals and their health staff, who then might promote infant formula or tolerate violations^(7,20)^. For example, a study in Hangzhou and Shenzhen cities revealed that around 60% of health workers recommended infant formula to mothers^(7)^. Vague statements about the benefits of infant formula might have been used to impose it as a ‘solution’ for vulnerable mothers (e.g., mental distress, pressure of feeding and caring for her child)^(41)^. Giving advice to feed the baby with infant formula might be an easy ‘solution’ for health staff who did not have enough time, knowledge and/or skills to support a mother to successfully breastfeed her infant^(41)^. A study in Wuhan revealed that only one of every five female physicians and nurses received any breast-feeding training and coaching after graduation and their knowledge was surprisingly poor^(42)^. Breast-feeding education, training and coaching programmes in both China and other countries have proven to be effective in improving knowledge and practice of health staff^(43,44)^. It is essential to build capacity of health staff to support mothers from pregnancy, during their hospital stay and after discharge.

Moreover, mothers who encountered breast-feeding difficulties during their hospital stay were more likely to receive advice to feed the baby with infant formula from hospitals, modern mass media as well as their family members, relatives or friends. Those mothers greatly needed support to overcome breast-feeding challenge. As discussed above, a lack of time, capacity and skills of health workers to support breast-feeding might explain the higher prevalence of receiving advice of feeding infant formula from hospitals. Advice of feeding infant formula may come from multiple sources through modern mass media. First, BMS manufacturers and distributors are using modern mass media to reach more mothers and fathers. The BMS manufacturers and distributors might still make use of existing traditional mass media in cities such as TV, radio, magazines and books. An adjusted logistic regression model showed that the prevalence of receiving advice to feed the baby with infant formula from modern mass media in big cities was as high as that in non-poor and poor rural counties but lower than that in small and medium cities. Predominant platforms of modern mass media were WeChat moments or QQ chats (50·6 %), maternal and baby product malls (49·4 %) and organisations’ or companies’ websites, WeChat or Weibo (37·8 %). A recent study also found many infant formula promotions on parenting apps in China^(15)^. Inappropriate promotion of BMS products through modern mass media is difficult to monitor and sanction compared with traditional mass media^(45,46)^. Specifically, mothers may get advice of infant formula feeding from social media. China has the world’s largest number of internet users and most active environment for social media^(47)^. Social media is a good channel in China as computers, tablets, smart phones and internet plans are accessible and affordable, as well as popular among members of younger generations^(48)^. Infant feeding advice from family members (especially spouses) and friends might shape the practices of the mothers directly or indirectly through daily interactions and use of social media. Thus, future interventions should include immediate social networks of mothers especially their family members^(49,50)^.

Third, since abolishment of *the Measure* in 2017, only the *Maternal and Infant Health Care Law* and its *Implementation Measures* ^(51)^ and the *Advertising Law* ^(52)^ have provisions regarding the regulation of the inappropriate marketing of BMS in China. There are still major gaps in these two laws compared with the requirements of *the Code* and in view of the actual needs for regulating the marketing of BMS in China. The current report from WHO, UNICEF and IBFAN shows that policies in China cover only few provisions of *the Code* and has big gaps in informational/educational materials, promotion to general public, promotion in health care facilities, engagement with health workers and systems, labelling, monitoring and enforcement^(24)^. China would benefit from strengthening policies and regulations in alignment with *the Code* because it would create a legal corridor for breast-feeding promotion, protection and supports in all settings^(4)^. For example, giving free sample and advice on using BMS in health facilities and point-of-sales would then become illegal and be regulated^(23,24)^. In addition to regulating traditional media, modern mass media should be regulated too^(4,46)^. To monitor contents in modern mass media, maximising the role of international and governmental organisations, civil society groups in report violations can be an effective measure^(4,46)^. Given BMS companies are using and sponsoring groups or individuals to use modern media channels to promote their products, media firms should ensure the contents are compliant to *the Code* and national regulations^(46,53)^.

### Strengths and limitations

Compared with previous studies, the current study has several strengths. Firstly, by using data from a large sample of mothers from various locations and of different socio-economic status, it provides updated information on the marketing of BMS in China and compares promotional tactics of BMS manufacturers and distributors across different locations. Secondly, limiting the sample to mothers of infants 0–5·9 months reduced recall bias of exposure to infant formula promotion. However, the current study also faced several limitations. Health systems in twelve sample sites selected in the first stage have comparatively higher levels of executive capacity, which may be correlated with their capacity to provide a better supporting environment for maternal and child health. Thus, cautions are needed when generalising results suggested in the current study nationally and comparing these results with existing literature. Second, responses of mothers may still suffer from recall bias and social desirability bias. Third, the cross-sectional nature of the current study might suggest the possible associations between the place of residence or socio-economic status with the exposure to infant formula promotion rather than confirm the association. However, the reverse association would be small: it is more likely that a BMS manufacturer or distributor targets a certain group of mothers than exposure to infant formula promotion changes the place of residence or socio-economic status of the mothers. Fourth, although we capture key information relating to BMS, the content of our questionnaire was not as exhaustive as the complete list of the WHO’s NetCode Assessment Module^(54)^. Nonetheless, we captured two main aspects of infant formula promotion: free samples and advice to feed the baby with infant formula. Also, several questions are left unanswered by the survey. For example, it is not clear who recommended the formula in the hospitals, whether they received antenatal and postnatal care at the same hospital where they deliver.

## Conclusions

In conclusion, the promotion of infant formula in the form of free samples and advice is common in surveyed areas in China. The promotion has targeted women from different socio-economic groups, place of residence and at various time points such as during pregnancy, delivery, postnatal period and beyond. The study findings would suggest the need to strengthen regulation and enforcement of *the Code* that restricts the promotion of BMS, especially within health facilities and through modern forms of media. The local government, health sector, media, civil groups, mothers and family members in less urbanised areas should also be prepared to protect recommended breast-feeding practices and against the negative effect of BMS promotion.

## Supplementary material

For supplementary material accompanying this paper visit https://doi.org/10.1017/S1368980020005364. click here to view supplementary material
