# Supplementary material for: Advice to use infant formula and free samples are common in both urban and rural areas in China: a cross-sectional survey
Source: Public Health Nutr. 2021 Jan 8;24(8):1977–88. doi: 10.1017/S1368980020005364 (PMC8145468; doi:10.1017/S1368980020005364)
Supplement: Supplementary file 1 [file S1368980020005364sup.zip › S1368980020005364sup001.pdf]

| 个人编码 |
| --- |

# 全国母乳喂养影响因素调查

婴儿姓名（NAME）：______

性别（SEX）： ①男 ②女

婴儿出生日期（BD）：______年____月___日（阳历） 月龄:________

省/自治区/直辖市: __________县/市/区：__________

乡镇/街道：____________

家庭住址：____________村/居委会

联系电话（TEL）：____________________

调查日期：______年_____月_____日

调查员：______________ 审核员：____________

中国疾病预防控制中心
营养与健康所
二〇一七年九月

## 1. 基本情况

<table>
<tr><td colspan="6">孩子父母的基本情况</td></tr>
<tr><td>与孩子的关系<br>A1</td><td>年龄（周岁）</td><td>民族<br>1 汉族 2 蒙古族 3 藏族 4 维吾尔族 5 苗族 6 彝族 7 回族 8 壮族 9 布依族 10 朝鲜族 11 满族 12 侗族 13 瑶族 14 白族 15 土家族 16 哈尼族 17 哈萨克族 18 傣族 19 黎族 20 其它</td><td>文化程度<br>1 未接受正规学校教育<br>2 小学未毕业<br>3 小学毕业<br>4 初中毕业<br>5 高中/中专/技校毕业<br>6 大专毕业<br>7 本科毕业<br>8 研究生及以上</td><td>职业<br>1 农林牧渔水利业生产人员<br>2 生产、运输设备操作人员及有关人员<br>3 商业、服务业人员<br>4 国家机关、党群组织、企事业单位负责人<br>5 办事人员和有关人员<br>6 专业技术人员<br>7 军人<br>8 其他劳动者<br>9 在校学生<br>10 未就业<br>11 家务</td><td>过去半年，平均一周与孩子共同居住几天？</td></tr>
<tr><td>父亲</td><td>□□<br>（A2a）</td><td>□□<br>（A3a）</td><td>□<br>（A4a）</td><td>□□<br>（A5a）</td><td>□<br>（A6a）</td></tr>
<tr><td>母亲</td><td>□□<br>（A2b）</td><td>□□<br>（A3b）</td><td>□<br>（A4b）</td><td>□□<br>（A5b）</td><td>□<br>（A6b）</td></tr>
<tr><td>A6c</td><td colspan="2">孩子的父亲现居住地是？</td><td colspan="3">1 非户籍地所在省<br>2 在户籍地所在省，但非户籍地所在市<br>3 在户籍地所在省、市，但非户籍地所在区/县<br>4 在户籍地所在省、市、区/县，但非户籍所在街道/乡镇<br>5 在户籍地所在省、市、区/县、街道/乡镇 （跳至 A7）<br>9 其他，____________________</td></tr>
</table>

| | | |
|---|---|---|
| A6d | 孩子的父亲在现居住地居住了多久？ | 1 ＜1 个月<br>2 1-5 个月<br>3 ≥6 个月 |
| A7 | 孩子的**母亲**现居住地是？ | 1 非户籍地所在省<br>2 在户籍地所在省，但非户籍地所在市<br>3 在户籍地所在省、市，但非户籍地所在区/县<br>4 在户籍地所在省、市、区/县，但非户籍所在街道/乡镇<br>5 在户籍地所在省、市、区/县、街道/乡镇 （跳至 B1）<br>9 其他，__________________ |
| A7a | 孩子母亲在现居住地居住多久？ | 1 ＜1 个月<br>2 1-5 个月<br>3 ≥6 个月 |

## 2. 母乳喂养相关情况

| | | |
|---|---|---|
| B1 | 婴儿的出生体重 | □□□□g（不清楚填 9999） |
| B3 | 婴儿的出生孕周：<br>（记录满的孕周数，例如 39 周+5 天，则记录 39 周） | □□周（跳至 B4）<br>9 不清楚 |
| B3a | 婴儿是否为早产儿？ | 1 是 2 否 9 不清楚 |
| B4 | 孩子出生后多久，开始吸吮乳房？<br>（指新生儿第一次吸吮母亲乳头的时间，婴儿试吃也算，不论是否真正吸吮到乳汁） | 1 _______ 小时<br>2 _______ 天<br>8 没吃过母乳<br>9 不记得/不知道 |
| B4a | 您认为产后多久应该开始让孩子吸吮乳房？ | _______小时 |
| B5 | 您知道产后几天内的乳汁为初乳？ | _______天<br>9 不知道 |

| | | |
|---|---|---|
| B5a | 您认为初乳的营养价值怎么样？<br>（可多选） | 1 初乳营养丰富，对孩子健康有益<br>2 初乳和普通乳汁没什么区别<br>3 初乳含有丰富的免疫活性成分<br>4 初乳没什么营养价值<br>9 不知道 |
| B6 | 您是产后几天开始感觉到乳房充盈（有奶或下奶）的？（不满 1 天记为 00，满 1 天不满 2 天记为 01，以此类推。） | ________天<br>88 一直没奶<br>99 不知道/没感觉 |
| B6a | 没下奶时，您是否坚持让孩子吸吮乳房？ | 1 是　2 否 |
| B6c | 您认为以下哪种是最有效的刺激乳汁分泌的方法？ | 1 婴儿吸吮<br>2 按摩、热敷、针灸<br>3 吃药（中药、西药）<br>4 多吃一些下奶的食物，多喝汤<br>5 其他 __________ |
| B7 | 您感觉孩子真正吃到的第一口母乳是在产后第几天？（不满 1 天记为 00，满 1 天不满 2 天记为 01，以此类推。） | 1 ________天<br>2 没吃过母乳<br>99 不知道/没感觉 |
| B8 | 孩子出生后是否喂过母乳？ | 1 是（跳至 B10）　2 否 |
| B9 | 没喂过母乳的原因是什么？(请选出导致以下行为最主要的原因，不超过 3 个）(完成此题,跳至 B15) | 1 婴儿配方奶粉更有营养<br>2 觉得母乳喂养麻烦或很累<br>3 担心母乳喂养影响身材或形象<br>4 母亲恢复工作或学业<br>5 母亲生病<br>6 无母乳<br>7 婴儿生病<br>8 婴儿拒绝吸吮<br>9 其他 ____________ |
| B10 | 过去 24 小时内，给孩子喂过母乳吗？ | 1 喂过（跳至 B13）<br>2 没喂过，但还没断母乳，仅过去 24 小时内没吃（继续回答 B11） |

| | | |
|---|---|---|
| | | 3 没喂过，孩子已断母乳，孩子断母乳的月龄是 B10a ________月龄（不清楚填 99）（跳至 B12） |
| B11 | 过去 24 小时内没喂母乳的原因是？(请选出最主要的原因，不超过 3 个)<br>(完成此题,跳至 B15) | 1 孩子大了，觉得该断奶了<br>2 孩子大了，母乳没什么营养了<br>3 觉得母乳喂养麻烦或很累<br>4 母亲恢复工作或学业或临时外出<br>5 母亲生病<br>6 婴儿生病<br>7 婴儿拒绝吸吮<br>8 其他 _____________ |
| B12 | 给孩子断母乳的最主要原因是什么？(请选出最主要的原因，不超过 3 个)<br>(完成此题,跳至 B15) | 1 孩子大了，觉得该断奶了<br>2 孩子大了，母乳没什么营养了<br>3 婴儿配方奶粉更有营养<br>4 觉得母乳喂养麻烦或很累<br>5 担心母乳喂养影响身材或形象<br>6 母亲恢复工作或学业或临时外出<br>7 母亲生病<br>8 母乳不够，不能满足婴儿需要；从______月龄开始觉得不够？<br>9 婴儿生病<br>10 婴儿拒绝吸吮<br>11 其他 _____________ |
| B13 | 过去 24 小时，您如何喂母乳？ | 1 直接哺乳 (跳至 B14)<br>2 直接哺乳+挤出乳汁喂养<br>3 每次都挤出乳汁喂养 |
| B13a | 为什么要挤出乳汁喂养？<br>(单选题,只选出最主要的 1 个原因即可) | 1 乳头结构异常（内陷等）<br>2 乳房乳头疼痛（皴裂、流血等）<br>3 婴儿出生缺陷（舌系带短等）<br>4 婴儿衔接不好，吃不到奶<br>5 母亲临时外出，不能按时喂哺<br>6 母亲恢复工作，不能按时喂哺 |

| | | |
|---|---|---|
| | | 7 月嫂/月子中心告知<br>8 乳头混淆<br>9 其他 |
| B14 | 过去 24 小时内，给孩子喂过几次母乳？ | ________次 |
| B15 | 过去 24 小时内，孩子是否用带奶嘴的奶瓶喝过水、婴儿配方奶等奶类或果汁等液体？ | 1 是　2 否 |
| B16 | 过去 24 小时内，孩子是否喝过白开水、矿泉水、纯净水或瓶装水？ | 1 是，喝过几次？_______<br>2 否 |
| B17 | 过去 24 小时内,孩子是否喝过婴儿配方奶？ | 1 是，喝过几次？_______<br>平均每次喝多少______毫升?<br>2 否 |
| B18 | 过去 24 小时内，孩子是否喝过牛奶、羊奶等鲜奶或普通奶粉？ | 1 是，喝过几次？_________<br>2 否 |
| B19 | 过去 24 小时内，孩子是否喝过酸奶? | 1 是　2 否 |
| B20 | 过去 24 小时内，孩子是否喝过糖水，果汁，茶或碳酸饮料？ | 1 是　2 否 |
| B21 | 过去 24 小时内，孩子是否喝过含乳饮料或植物饮料如豆奶、核桃露等？ | 1 是　2 否 |
| B22 | 过去 24 小时内，孩子是否喝过稀粥？ | 1 是　2 否 |
| B23 | 过去 24 小时内，孩子是否吃过肉汤或菜汤（喝过或者蘸着吃过都算）？ | 1 是　2 否 |
| B24 | 过去 24 小时内，孩子是否吃过营养素补充剂？（例如：伊可新、钙片、鱼肝油、维生素等） | 1 是　2 否 |
| B25 | 过去 24 小时内，孩子是否已经添加固体、半固体或糊状食物(即辅食,不包括上面题目中已经问过的液体)？ | 1 是　2 否（跳至 B27） |

| | | |
|---|---|---|
| B26 | 过去 24 小时内，孩子吃过几餐固体、半固体或糊状食物（填次数，包括正餐和零食，除外液体）？ | _____餐 |
| B27 | 请问您最早是在什么时候考虑如何喂养孩子的？ | 1 孕前<br>2 孕早期<br>3 孕中期<br>4 孕晚期<br>5 分娩后 |
| B28a | 请问第一次给孩子喂母乳以外的食物是什么？（包括：水、饮料、婴儿配方奶粉或其他任何食物，但不包括药物和营养素补充剂） | _____<br>9 至今没添加过母乳以外的食物（跳至 B29） |
| B28b | 第一次给孩子喂母乳以外的水、饮料、婴儿配方奶粉或其他食物是孩子多大的时候？ | 1 _____日龄<br>2 _____月龄 |
| B28c | 第 1 次给孩子喂母乳以外的水、饮料、配方奶粉或其他食物，最主要的原因是什么，不超过 3 个？（多选） | 1 亲友医生等建议<br>2 婴儿需要增加液体摄入<br>3 婴儿配方奶粉有营养<br>4 收到赠送的婴儿配方奶粉<br>5 觉得母乳喂养麻烦或很累<br>6 担心母乳喂养影响身材或形象<br>7 母乳不够，不能满足婴儿需要<br>6 遇到哺乳困难（乳腺炎、乳头疼痛等）<br>8 认为孩子到了该添加辅食的年龄<br>9 孩子对其他食物感兴趣<br>10 需要和孩子分开<br>11 其他：_______________ |
| B29 | 您认为婴儿几月龄内应该只吃母乳，不应该添加任何其它食物或液体（包括水、婴儿配方奶、其它奶和固体/半固体食物）？ | □□（不知道填 99） |

| | | |
|---|---|---|
| B30 | 您认为母乳喂养应该持续到孩子多大（月龄）？ | □□（不知道填 99） |
| B31 | 您认为如何母乳喂养好？ | 1 按需喂母乳（孩子想吃或母亲想喂，就可以喂母乳）<br>2 按时喂母乳<br>3 无所谓<br>9 不知道 |
| B32 | 月子期间，您的孩子是否出现过下列情况？ | 1 只吃妈妈的乳头不吃奶嘴<br>2 只吃奶嘴不吃妈妈乳头<br>3 以上两种情况都出现过<br>4 妈妈乳头和奶嘴都吃<br>5 月子期间孩子没用过奶嘴 |
| B33 | 您认为母乳喂养的好处是什么？<br>（可多选） | 1 可促进母体子宫等器官恢复<br>2 有利于母亲身材恢复、减轻体重<br>3 有利于降低母亲乳腺癌、卵巢癌的发病率<br>4 可以延迟月经恢复<br>5 可以增进母子情感交流<br>6 更经济实惠、更安全<br>7 母乳能满足婴儿不同时期的生理需要，这是其他代乳品都不能比拟的<br>8 可以提高孩子免疫力，降低感染性疾病的发生<br>9 有利于孩子的智力和情感发育<br>10 可能降低孩子长大后发生超重肥胖和慢性病的风险<br>11 孩子不容易过敏 |

## 3. 母婴健康状况

| | | |
|---|---|---|
| C1 | 被调查的孩子是您的第几个孩子？ | □，如回答第 1 个，跳至 C6 |
| C2 | 您是否母乳喂养过上个孩子？ | 1 是<br>2 否（跳至 C6） |
| C3 | 您母乳喂养上个孩子到多大？ | ______月 |
| C6 | 本次怀孕,您是否被诊断过妊娠期糖尿病？ | 1 是　　2 否 |
| C7 | 您是否被诊断过妊娠高血压疾病？ | 1 是　　2 否 |
| C8 | 您的身高是多少厘米？ (记不清填 999)？ | _____ 厘米 |
| C9 | 您本次怀孕前体重是多少公斤？(结果保留 1 位小数，记不清填 999.9) | _____ 公斤 |
| C11 | 您怀孕前所用胸罩罩杯的大小是多大？ | ①AA　②A　③B　④C ⑤D<br>⑥E　⑦F　⑨不清楚 |
| C13 | 您目前是否已经恢复月经？ | 1 是（跳至 C15）<br>2 否 |
| C14 | 如果还未恢复，请问恢复月经后，您是否还打算继续母乳喂养？　**（答完此题，跳至 C17）** | 1 是<br>2 否，为什么？__________<br>8 已经断奶了<br>9 没想好 |

| | | |
|---|---|---|
| C15 | 这次分娩完，产后多久恢复月经的？ | _______月 |
| C16 | 请问恢复月经后，您是否还在继续母乳喂养？ | 1 是<br>2 否，为什么？__________<br>8 恢复月经前就断奶了 |
| C17 | 孩子出生后一个月内是否因病住过院？ | 1 是 2 否 |
| C18 | 孩子出生后是否诊断过新生儿低血糖？ | 1 是 2 否 |
| C19 | 孩子出生后是否诊断过新生儿黄疸？ | 1 是 2 否 |
| C20 | 过去 2 周内，您的孩子是否患过腹泻？ | 1 是 2 否 |
| C21 | 过去 2 周内，您的孩子是否得过呼吸系统疾病（如感冒、气管炎、支气管炎、肺炎等）？ | 1 是 2 否 |
| C22 | 孩子出生到现在，您或家人是否担心过孩子的体重长得过快或过慢？ | 1 是，担心孩子体重增长过快（跳至 C22b）<br>2 是，担心孩子体重增长过慢<br>3 不担心 （跳至 D1） |
| C22a | 由于担心体重增长过慢，您是如何调整婴儿喂养？（答完此题，跳至 D1） | 1 增加母乳喂养频次或时间<br>2 妈妈增加食物摄入<br>3 添加婴儿配方奶粉<br>4 添加辅食<br>5 未调整 |
| C22b | 由于担心体重增长过快，您是如何调整婴儿喂养？ | 1 减少母乳喂养频次或缩短哺乳时间<br>2 妈妈减少食物摄入<br>3 减少婴儿配方奶粉<br>4 未调整 |

## 4. 家庭及社区对母乳喂养的支持

| | | |
|---|---|---|
| D1 | 月子期间，您最主要由谁照顾：（单选） | 1 丈夫<br>2 母亲<br>3 婆婆<br>4 父亲/公公<br>5 月嫂<br>6 普通保姆<br>7 月子中心工作人员<br>8 其他__________ |
| D2 | 月子期间，您的孩子最主要由谁照顾：（单选） | 1 孩子母亲<br>2 孩子父亲<br>3 孩子祖母<br>4 孩子外祖母<br>5 孩子祖父/外祖父<br>6 月嫂<br>7 普通保姆<br>8 月子中心工作人员<br>9 其他__________ |
| D3 | 月子期间，您是否有不愿意哺乳的时候？ | 1 从未（跳至 D4a）<br>2 极少（跳至 D4a）<br>3 有时<br>4 经常<br>5 始终 |
| D3a | 月子期间，您不愿意哺乳的原因是？ | 1 分娩疲惫或分娩疼痛<br>2 乳头皲裂或疼痛<br>3 母乳不足<br>4 乳头内陷<br>5 母乳喂养姿势掌握不好<br>6 心情不好<br>7 哺乳频繁<br>8 睡眠不够<br>9 想尽快恢复身材，节食<br>10 其他，___________ |

| | | | | | | | |
|---|---|---|---|---|---|---|---|
| 您周围人赞同以下哪种说法？<br>1:母乳比婴儿配方奶粉好　2：虽然母乳好，但配方粉可以接受　3：母乳与婴儿配方奶粉一样好　4：婴儿配方奶粉比母乳好　8 不适用　9 不知道 | | | | | | | |
| D4a | 您的母亲 | 1 | 2 | 3 | 4 | 8 | 9 |
| D4b | 您的婆婆 | 1 | 2 | 3 | 4 | 8 | 9 |
| D4c | 您的丈夫 | 1 | 2 | 3 | 4 | 8 | 9 |
| D4d | 您最要好的朋友 (只选择对您影响最大的一个好友对喂养方面的态度) | 1 | 2 | 3 | 4 | 8 | 9 |
| D4e | 您的月嫂或月子中心工作人员 | 1 | 2 | 3 | 4 | 8 | 9 |
| D4f | 您的保姆 | 1 | 2 | 3 | 4 | 8 | 9 |
| D4g | 医务人员（只选择对您影响最大的一位） | 1 | 2 | 3 | 4 | 8 | 9 |
| D4h | 护工 | 1 | 2 | 3 | 4 | 8 | 9 |
| D5 | 您小时候，您的母亲如何喂养您？ | 1 母乳喂养<br>2 婴儿配方奶/牛奶喂养<br>3 母乳和婴儿配方奶/牛奶混合喂养<br>4 其他<br>9 不知道 | | | | | |
| D6 | 月子期间，您是否请了月嫂或去了月子中心疗养？ | 1 都没有（跳至 D10）<br>2 是，请了月嫂<br>3 是，去了月子中心<br>4 是，既请了月嫂又去了月子中心 | | | | | |
| D7 | 月嫂或月子中心是否提供母乳喂养宣传和教育（宣传或教育材料）？ | 1 是　　2 否 | | | | | |
| D8 | 月嫂或月子中心是否指导产妇如何母乳喂养？ | 1 是　　2 否 | | | | | |
| D9 | 请评价月嫂或月子中心对您自身喂养行为的影响程度： | 1 影响非常大，直接影响了孩子日后的喂养方式<br>2 有一定的影响<br>3 一般，没什么影响 | | | | | |
| D9a | 月嫂或月子中心是否曾经建议您添加婴儿配方奶粉？ | 1 是　　2 否 | | | | | |

| | | |
|---|---|---|
| D10 | 您是否参加过妈妈小组(如妈妈微信群,APP妈妈群，线下妈妈组等)？ | 1 是 2 否（跳至 E1） |
| D10a | 请评价您参加的妈妈小组对母乳喂养行为的态度： | 1 十分支持母乳喂养 2 支持母乳喂养 3 中立 4 支持配方奶粉喂养<br>5 十分支持配方奶粉喂养 |
| D10b | 请评价您参加的妈妈小组对您自身喂养行为的影响程度： | 1 影响非常大，直接影响了孩子日后的喂养方式<br>2 有一定的影响<br>3 一般，没什么影响 |
| D10c | 您是否从妈妈小组收到过添加婴儿配方奶粉的建议？ | 1 是 2 否 |

## 5. 卫生服务状况

| | | |
|---|---|---|
| E1 | 本次怀孕，您是否参加过孕期保健(产前检查)？ | 1 是 2 否（跳至 E7） |
| E2a | 孕期保健机构(妇幼保健院/医院/乡镇卫生院/社区卫生服务中心）是否开展了母乳喂养教育？ | 1 是，开展了<br>2 没有开展（跳至 E7）<br>3 不知道（跳至 E7） |
| E2 | 孕期保健机构(妇幼保健院/医院/乡镇卫生院/社区卫生服务中心）中谁负责开展母乳喂养教育？(多选) | 1 医生<br>2 护士<br>3 助产士<br>4 哺乳顾问<br>5 其他 |
| E3 | 您是否参加过孕期保健机构的母乳喂养教育？ | 1 是 2 否（跳至 E7） |
| E6 | 您觉得孕期保健机构提供的母乳喂养教育对您的母乳喂养有帮助吗? | 1 有帮助 2 没有帮助 |
| E7 | 本次分娩，您采用的是哪种方式？ | 1 自然分娩<br>2 人工辅助分娩（侧切、使用吸引、产钳等）<br>3 剖宫产 |

| | | |
|---|---|---|
| E7b | 本次分娩后 24 小时内，您是否出现大出血？（出血量大于 500 毫升） | 1 是 2 否 9 不知道 |
| E8 | 您是在哪分娩的？ | 1 省级（直辖市）及以上保健院或医院<br>2 地市级保健院或医院<br>3 县级保健院或医院<br>4 乡镇卫生院<br>5 村卫生室<br>6 家 （跳至 F1）<br>7 私立医院<br>8 其他 |
| E9 | 您分娩时在医院住了多少天？（不满 1 天记为 00，满 1 天不满 2 天记为 01，以此类推。） | 住院_____天<br>99. 没住院 （跳至 F1） |
| E10 | 您在住院分娩期间，谁陪床？ （单选） | 1 护工<br>2 丈夫<br>3 其他家属<br>4 没有陪床 |
| E11 | 您在分娩住院期间，是否有过哺乳困难（乳头皲裂、乳头异常、乳房胀痛、乳腺管堵塞、乳腺炎等，有过其中一种就算）？ | 1 是 2 否（跳至 E12） |
| E11a | 住院期间遇到的哺乳困难，您到哪里寻求的帮助？（多选题） | 1 妈妈或婆婆<br>2 亲戚或朋友<br>3 专业医护人员<br>4 病房护工<br>5 通乳师<br>6 自己<br>7 其他 |
| E11c | 住院期间遇到的哺乳困难，最终您是否得到了解决？ | 1 是 2 否 |
| E11d | 您感觉这些困难是否对您日后的母乳喂养造成了一定的阻碍？ | 1 是 2 否 |

| | | |
|---|---|---|
| E12 | 您觉得分娩住院期间医院提供的母乳喂养宣传、教育、指导等对您有帮助吗? | 1 有帮助　2 没有帮助<br>3 没提供 |
| E13 | 医院是否向您推荐了出院后可以寻求的支持母乳喂养组织? | 1 推荐了，是哪里？______<br>2 否（跳至 E14） |
| E13a | 出院后您是否向这些组织寻求过母乳喂养的帮助? | 1 是<br>2 否，为什么？____ |
| E14 | 出院后，您所在社区 / 乡镇的卫生服务中心或分娩医院是否提供了产后访视、远程指导等服务? | 1 是　2 否 |
| E16 | 出院后，您是否发生过乳头皲裂、乳头异常、乳房胀痛、乳腺管堵塞、乳腺炎等症状? | 1 有<br>2 没有（跳 F1） |
| E17 | 出院后在出现乳头皲裂、乳头异常、乳房胀痛、乳腺管堵塞、乳腺炎等症状时，您到哪里寻求的帮助？（多选） | 1 自己的家人<br>2 亲戚或朋友<br>3 专业医护人员<br>4 月嫂或月子中心人员<br>5 催奶师（通乳师或母乳喂养师）<br>6 婴儿配方粉生产销售人员<br>7 传统医学/按摩<br>8 自己<br>9 其他 |
| E17a | 出院后，您在网上通过什么平台寻求解决以上困难的? | 1 母婴用品商城<br>2 医生及医院的网站及平台<br>3 科普类网站、微信公众号以及微博平台<br>4 微信朋友圈、QQ 好友的交流<br>5 网络电视剧及视频<br>6 没有通过网络寻求帮助<br>7 其他 |
| E18 | 出院后遇到的哺乳困难，最终您是否得到了解决? | 1 是　2 否 |
| E19 | 您感觉这些困难是否对您日后的母乳喂养造成了一定的阻碍? | 1 是　2 否 |

## 6. 工作场所

| | | |
|---|---|---|
| F1 | 您分娩前的职业类型属于下面哪种类型？ | 1 正规就业<br>2 非正规就业（跳至 F18） |
| F2 | 您是否有带薪产假？ | 1 是　　2 否（跳至 F9） |
| F3 | 您单位考勤制度中规定产后带薪休假最多可以休多少天？（包括国家规定的产假和单位规定可以休的延长产假的总和） | _____ 天 |
| F5 | 您产假期间的收入大约相当于上班期间的百分之多少？（平均值） | 1　______%<br>9　不清楚 |
| F6 | 您目前是否已经恢复工作？ | 1 是　　2 否（跳至 F10） |
| F7 | 您实际休了多少天产假？ | _____ 天 |
| F7a | 如果 F3≥F7 即规定带薪产假时间≥实际休的产假时间，为什么？ | 1 产假时间与实际休的时间相等<br>2 扣钱扣得比较多，提前恢复工作<br>3 实际工作需要，提前恢复工作<br>4 家中有人带孩子，提前恢复工作<br>5 其他 |
| F7b | 如果 F3<F7 即规定带薪产假时间<实际休的产假时间，为什么？ | 1 坚持母乳喂养，休了别的假<br>2 家中没人带孩子，休了别的假<br>5 其他 |
| F9 | 恢复工作后，您如何继续母乳喂养婴儿？（回答完此题跳到 F11） | 1 在单位（工作场所）喂奶<br>2 往返家和单位亲自喂奶<br>3 用吸奶器/手挤背奶回家<br>4 仅下班后回家喂奶<br>5 上班就断奶了 |
| F10 | 如目前尚未恢复工作，恢复工作后您打算如何继续母乳喂养？ | 1 在单位（工作场所）喂奶<br>2 往返家和单位亲自喂奶<br>3 用吸奶器/手挤背奶回家 |

| | | |
|---|---|---|
| | | 4 仅下班后回家喂奶<br>5 上班就断奶了<br>9 没想好 |
| F11 | 您从单位（或工作场所）到家采用平常交通工具，通常需要多长时间？ | _____ 分钟 |
| F12 | 您单位（或工作场所）母乳喂养的时间是如何规定的？ | 1 没有母乳喂养时间<br>2 工作日每天 1 个小时<br>3 其他 _____ |
| F13 | 您单位（或工作场所）是否设立了专用的母乳喂养室或哺乳室？ | 1 是 （跳至 F15） 2 否 |
| F14 | 您单位（或工作场所）是否有私密空间供吸乳或哺乳？ | 1 是，有独立办公室可供吸乳或哺乳（跳至 F15）<br>2 是，可以去卫生间进行吸乳（跳至 F15）<br>3 是，有其他空屋子（跳至 F15）<br>4 否 |
| F14a | 如果您需要吸乳或哺乳时怎么办？ | 1 躲在办公桌下<br>2 简单遮挡后在公共场合吸乳或哺乳<br>3 直接在公共场合吸乳或哺乳<br>4 断奶了<br>5 其他_____________ |
| F15 | 您单位（或工作场所）是否有能存放母乳的冰箱？ | 1 是 2 否 |
| F16 | 您的上司或单位领导是否支持母乳喂养？ | 1 支持 2 中立 3 不支持 4 不清楚 |
| F17 | 单位（或工作场所）同事中母乳喂养的比例？ | 1 多数母乳喂养<br>2 母乳和婴儿配方奶粉喂养各半<br>3 多数婴儿配方奶粉喂养 |
| F18 | 产后您打算或实际休息了多长时间？ | _____ 天 |

## 7. 社会环境及文化

| | | |
|---|---|---|
| G1 | 您是否接受过鼓励母乳喂养方面的信息？ | 1 是　 2　否（跳至 G5） |
| G3a | 您从哪里获得母乳喂养方面的信息？<br>（可多选） | 1 妇幼保健机构/分娩医院<br>2 传统媒体（电视/广播/杂志/书）<br>3 互联网（网站/微博/微信）<br>4 短信<br>5 其他_______________ |
| G3c | 您从哪些人中获得过母乳喂养方面的信息？<br>（多选） | 1 自己的家人<br>2 亲戚或朋友<br>3 专业医护人员<br>4 月嫂或月子中心人员<br>5 催奶师<br>6 婴儿配方粉生产销售人员<br>7 医院护工<br>8 其他 |
| G3d | 您获得过哪些母乳喂养信息？<br>（多选） | 1 母乳喂养好处<br>2 纯母乳喂养相关知识<br>3 母乳喂养姿势<br>4 母乳喂养时间<br>5 判断泌乳量充足的方法<br>6 其他 |
| G4 | 您觉得您接收到的母乳喂养相关信息在喂养孩子的实践中是否有用？ | 1 没用<br>2 大部分没用<br>3 大部分有用<br>4 有用<br>9 不知道 |
| G5 | 您是否接收过给孩子喂婴儿配方奶粉的建议？ | 1 是<br>2 否（跳至 G8） |

| | | |
|---|---|---|
| G7 | 您从哪里得到给孩子喂婴儿配方奶粉的建议？（多选） | 1 妇幼保健机构/分娩医院<br>2 传统媒体（电视/广播/杂志/书）<br>3 互联网（网站/微博/微信）（选此选项继续询问 G7a）<br>4 短信<br>5 其他________________ |
| G7a | 您在网上通过什么平台获得婴儿配方奶粉的喂养建议？（多选） | 1 母婴用品商城<br>2 医生及医院的网站及平台<br>3 科普类网站、微信公众号以及微博平台<br>4 微信朋友圈、QQ 好友的交流<br>5 网络电视剧及视频<br>6. 其他 |
| G8 | 您是否接收过配方奶粉免费赠品（包括孕妇乳母配方粉和婴幼儿配方粉）？ | 1 是<br>2 否（跳至 G10） |
| G9 | 您在哪个时期接收过婴儿配方奶粉免费赠品？（可多选） | 1 孕期________月份<br>2 分娩住院期<br>3 出院后<br>4 其他 |
| G10 | 根据您的经历，在公共场所，您认为是否需要母乳哺育室? | 1 是　　2 否 |
| G11 | 在您生活的区域内，您认为母乳哺育室是否充足？ | 1 是　　2 否 |
| G12 | 您是否曾有过因在公共场所哺乳不便而减少外出的经历? | 1 是　　2 否 |
| G13 | 您是否曾有过因在公共场所哺乳不便而给孩子喂婴儿配方奶粉的经历? | 1 是　　2 否 |
| G14 | 请评价下列说法是否符合您的状况？ 1 是 2 否 | |
| | 在公共场合进行母乳喂养时，我觉得尴尬。<br>在女性亲戚和朋友面前进行母乳喂养，我觉得尴尬。<br>在男性亲戚和朋友面前进行母乳喂养，我觉得尴尬。 | 1 是　　2 否<br>1 是　　2 否<br>1 是　　2 否 |

## 8.家庭经济状况

| | | |
|---|---|---|
| K1 | 2016 年，您家的家庭总收入是多少？ | 1 □□□□□□□元/年 A8a<br>2 拒绝回答<br>9 不清楚 |
| K2 | 2016 年，您家的总支出是多少？ | 1 □□□□□□□元/年 A9a<br>2 拒绝回答<br>9 不清楚 |
| K3 | 上月您家用于食物的支出是多少？ | 1 □□□□□□□元/月 A10a<br>2 拒绝回答<br>9 不清楚 |
| K4 | 上月您家用于婴儿配方奶粉的支出是多少？ | 1 □□□□元/月<br>2 没有使用婴儿配方粉<br>3 拒绝回答<br>9 不清楚 |
| K5 | 您全家一共有几口人？（包括所有孩子在内的具有共同经济收入和支出的家庭成员） | □□ |

调查员签字：____________　　审核员签字：___________
