# Supplementary material for: Advice to use infant formula and free samples are common in both urban and rural areas in China: a cross-sectional survey
Source: Public Health Nutr. 2021 Jan 8;24(8):1977–88. doi: 10.1017/S1368980020005364 (PMC8145468; doi:10.1017/S1368980020005364)
Supplement: Supplementary file 1 [file S1368980020005364sup.zip › S1368980020005364sup002.pdf]

Code

National Survey on Factors Influencing Breastfeeding in China

Baby's Name:____________________

Sex: (1) Boy (2) Girl

Date of Birth (BD): _________(yyyy)________(mm)_________(dd) (Solar Calendar)

Age: _____________________months

Province/Autonomous Region/Municipality:____________________

County/City/District:_______________

Town/Street:_____________________

Address: ____________________Village/Neighborhood Committee

Tel.: ____________________________________

Date of Survey: ______(yyyy)___________(mm)____(dd)

Surveyed by:__________________Reviewed by:_____________

Chinese Center for Disease Control and Prevention

Institute for Nutrition and Health

September 2017

1. Basic Information

Parents' Basic Information					
**Relationship**<br>A1	**Age**	**Ethnic Group**<br>1 Han<br>2 Mongolian<br>3 Tibetan<br>4 Uygur<br>5 Miao<br>6 Yi<br>7 Hui<br>8 Zhuang<br>9 Bouyei<br>10 Korean<br>11 Manchu<br>12 Dong<br>13 Yao<br>14 Bai<br>15 Tujia<br>16 Hani<br>17 Kazak<br>18 Dai<br>19 Li<br>20 Others	**Educational Background**<br>1. No formal school education<br>2. Failed to graduate from elementary school<br>3. Graduated from elementary school<br>4. Graduated from junior high school<br>5. Graduated from senior high school /technical secondary school<br>6. Graduated from college<br>7. Bachelor<br>8. Master or above	**Occupation**<br>1. Agriculture, forestry, animal husbandry, fishery and water conservancy practitioner<br>2. Production and transportation equipment operators and related personnel<br>3. Business and service industry practitioner<br>4. Head of State authorities, party group organizations, enterprises and institutions<br>5. Clerk and related personnel<br>6. Professional technician<br>7. Soldier<br>8. Other workers<br>9. Student<br>10. Unemployed<br>11. Housework	**How many days in a week did you live together with your baby on average in the past 6 months?**
**Father**	□□<br>(A2a)	□□<br>(A3a)	□<br>(A4a)	□□<br>(A5a)	□<br>(A6a)
**Mother**	□□<br>(A2b)	□□<br>(A3b)	□<br>(A4b)	□□<br>(A5b)	□<br>(A6b)
A6c	**Father**'s current place of residence		1. Not in the province of registered residence<br>2. In the province of registered residence, but not in the city of registered residence<br>3. In the province/city of registered residence, but not in the district/county of registered residence<br>4. In the province/city/district/county of registered residence, but not in the street/town/township of registered residence<br>5. In the province/city/district/county/street/town/township of registered residence (go to A7)<br>9. Others		

A6d	How long has the baby's father lived in the current place of residence?	1. ＜1 month<br>2. 1-5 months<br>3. ≥6 months
A7	**Mother**'s current place of residence	1. Not in the province of registered residence<br>2. In the province of registered residence, but not in the city of registered residence<br>3. In the province/city of registered residence, but not in the district/county of registered residence<br>4. In the province/city/district/county of registered residence, but not in the street/town/township of registered residence<br>5. In the province/city/district/county/street/town/township of registered residence (go to B1)<br>9. Others, __________
A7a	How long has the baby's mother lived in the current place of residence?	1. ＜1 month<br>2. 1-5 months<br>3. ≥6 months

2. About Breastfeeding

B1	Your baby's weight at birth	□□□□g (or "9999" if not sure)
B3	Your gestational age at birth<br>(take only full weeks, e.g. 39w+5d = 39w)	□□ weeks (go to B4)<br>9. Not sure
B3a	Is it a preterm birth?	1. Yes<br>2. No<br>9. Not sure
B4	How soon after delivery did your baby latch on to the breast?<br>(When the newborn baby latched on, regardless whether she/he really sucked and the milk came in.)	1. _________hours<br>2. _________days<br>8. Never breastfed<br>9. Forgot/Not sure
B4a	How soon after delivery do you think is the proper time for the first latch?	_________hours
B5	Do you know for how many days after delivery moms produce colostrum?	_________days<br>9. No idea
B5a	Do you think colostrum is nutritious? (Multiple choices)	1. It's nutrient-rich and beneficial to infants.<br>2. It's much the same as milk.<br>3. It's full of immune-boosting factors.<br>4. It's of little nutritional value.<br>9. No idea.

B6	On what day after delivery did your breasts fill up with milk?<br>("00" if less than one full day; "01" if less than two full days; etc.)	_________days<br>88. Never<br>99. Not sure
B6a	Did you keep your baby practicing latches before milk came in?	1. Yes<br>2. No
B6c	In your opinion, what is the best way to stimulate milk production?	1. Baby sucking<br>2. Massage, hot patch, acupuncture & moxibustion<br>3. Medicines (Western and Chinese)<br>4. Food and soup that may help increase milk supply<br>5. Others _____________
B7	On what day after delivery did your baby have his/her first taste of milk?<br>("00" if less than one full day; "01" if less than two full days; etc.)	1. _______days<br>2. Never<br>99. Not sure
B8	Did you ever breastfeed your baby after birth?	1. Yes (go to B10)<br>2. No
B9	Why did you choose not to breastfeed? (Choose no more than three of the main reasons, then go to B15)	1. Infant formula is more nutritious than breast milk.<br>2. Breastfeeding is hard or painful.<br>3. There might be significant changes in body shape.<br>4. Mother has gone back to work/school.<br>5. Mother was sick.<br>6. No milk came in.<br>7. The baby was sick.<br>8. The baby refused to suckle.<br>9. Others______
B10	Did you breastfeed your baby in the past 24 hours?	1. Yes (go to B13)<br>2. No, but with no intend to wean the baby.<br>3. No, the baby has been weaned at B10a_ _______months old.<br>"99" if not sure (go to B12)
B11	Why didn't you breastfeed in the past 24 hours? (Choose no more than three of the main reasons, then go to B15)	1. It was time to wean the baby.<br>2. Breast milk is no longer nutritious for a baby at this age.<br>3. Breastfeeding is hard or painful.

		4. Mother has gone back to work/school or had a temporary going-out.<br>5. Mother was sick.<br>6. The baby was sick.<br>7. The baby refused to suckle.<br>8. Others______
B12	Why did you wean your baby? (Choose no more than three of the main reasons, then go to B15)	1. It was time to wean the baby.<br>2. Breast milk is no longer nutritious for a baby at this age.<br>3. Infant formula is more nutritious than breast milk.<br>4. Breastfeeding is hard or painful.<br>5. There might be significant changes in body shape.<br>6. Mother has gone back to work/school or had a temporary going-out.<br>7. Mother was sick.<br>8. Low milk supply since__months old.<br>9. The baby was sick.<br>10. The baby refused to suckle.<br>11. Others______
B13	How did you give breast milk in the past 24 hours?	1. Breastfed exclusively (go to B14)<br>2. Breastfed + pumped milk for a bottle<br>3. Only pumped and bottle-fed
B13a	Why did you pump and bottle-feed? (Single choice, choose only one of the most important reasons)	1. Nipple abnormalities (retraction, etc.)<br>2. Nipple soreness (cracks, bleeding, etc. ）<br>3. Birth defects (tight tongue frenulum, etc.)<br>4. Poor latching<br>5. Mother had a temporary going-out and couldn’t breastfeed on time<br>6. Mother has gone back to work and made regular breastfeeding impossible<br>7. Advised by postnatal caregivers/care center staff<br>8. Nipple confusion<br>9. Others
B14	How many times in the past 24 hours did you give breast milk?	______________times
B15	Was your baby given water, infant formula or other fluids (milk, juice, etc.) in the past 24 hours through bottle nipples?	1. Yes<br>2. No
B16	Was your baby supplemented with boiled water, mineral water, purified water or bottled water in the past 24 hours?	1. Yes, ________times<br>2. No

B17	Was your baby given infant formula in the past 24 hours?	1. Yes, ________times at ________ ________ ml each on average<br>2. No
B18	Was your baby given cow's milk, ewes' milk or other fresh milk, or regular milk powders?	1. Yes, ________times<br>2. No
B19	Was your baby given yogurt in the past 24 hours?	1. Yes<br>2. No
B20	Was your baby given sweetened water, juice, tea or soda in the past 24 hours?	1. Yes<br>2. No
B21	Was your baby given milk beverage or botanical drinks (soy milk, walnut soup) in the past 24 hours?	1. Yes<br>2. No
B22	Was your baby given gruel in the past 24 hours?	1. Yes<br>2. No
B23	Did your baby drink or dip meat soup or vegetable soup in the past 24 hours?	1. Yes<br>2. No
B24	Was your baby given nutritional supplements (e.g. Vitamin A&D Drops, calcium tablets, cod liver oil, and vitamins) in the past 24 hours?	1. Yes<br>2. No
B25	Was your baby given solid, semi-solid or pasty food (i.e. complementary food, excluding fluids mentioned above) in the past 24 hours?	1. Yes<br>2. No (go to B27)
B26	How many solid, semi-solid or pasty meals has your baby had (meals and snacks, not including fluids) in the past 24 hours?	________meals
B27	When did you start thinking about the way to feed your baby?	1. Pre-pregnancy<br>2. First trimester<br>3. Second trimester<br>4. Third trimester<br>5. After childbirth
B28a	What was the first food that your baby had other than breast milk (water, beverage, infant formula and any other food, excluding medicine and nutritional supplements)?	_______<br>9. Hasn't had any yet (go to B29).
B28b	At which age was your baby first given water, beverage, infant formula or any food other than breast milk?	1________days<br>2________months
B28c	Why did you for the first time give your baby water, beverage, infant formula or any food other than breast milk? (Multiple choices: choose no more than three of the main reasons)	1. Advised by doctors, friends, family members.<br>2. The baby needed to increase fluid intake.<br>3. Infant formula is nutritious.<br>4. Received infant formula as a gift.<br>5. Breastfeeding is hard or painful.

		6. There might be significant changes in body shape.<br>7. Low milk supply<br>8. Encountered breastfeeding difficulties (mastitis, nipple soreness, etc.)<br>9. The baby was old enough to have complementary food.<br>10. The baby showed interests in other food.<br>11. The baby had to be separated from the mother.<br>12. Others____________________
B29	Till which month in your opinion shall baby be given no other food or fluid (including water, infant formula, other milk, solid or semisolid supplements) than breast milk?	☐☐ ("99" if no idea)
B30	Till which month shall baby be breastfed?	☐☐ ("99" if no idea)
B31	What is the proper way of breastfeeding?	1. Breastfeed the baby on demand (of mom or baby)<br>2. Follow regular fixed feeding time<br>3. It makes no difference to me<br>9. I don't know
B32	Which of the following situations applied to your baby when you were under postnatal care?	1. Only accepted breasts, refused bottle teats<br>2. Only accepted bottle teats, refused breasts<br>3. Both 1 and 2<br>4. Accepted breasts and bottle teats<br>5. Never tried bottle teats when mom was under postnatal care
B33	What do you think are the benefits of breastfeeding? (Multiple choices)	1. Help uterus and other organs return to normal<br>2. Help the mom regain a desired figure and reduce weight<br>3. Lower ovarian and breast cancer risk<br>4. Delay menstruation<br>5. Emotional connection and closeness between baby and mom<br>6. It's more economical and safer.<br>7. Breast milk can meet the baby's physiological needs at different stages, while other substitutes can't.<br>8. Build up immunity and reduce the risk of infectious diseases<br>9. Promote baby's emotional and intellectual development.

		10. Reduce the risk of overweight, obesity and chronic disease when the baby grows up.<br>11. Less likely to get an allergy

3. Maternal and child health

C1	This is your (first/second/third…) biological child.	□, go to C6 if the answer is "1"
C2	Did you breastfeed your previous baby?	1. Yes<br>2. No (go to C6)
C3	When did you have your previous baby weaned?	At ______months old
C6	Were you diagnosed with gestational diabetes during this pregnancy?	1. Yes<br>2. No
C7	Were you ever diagnosed with high blood pressure during pregnancy?	1. Yes<br>2. No
C8	What is your height? ("999" if not sure)	_______cm
C9	How much did you weight before this pregnancy? (to one decimal; "999.9" if not sure)	_______kg
C11	What was your bra cup size prior to pregnancy?	①AA ②A ③B ④C ⑤D<br>⑥E ⑦F ⑨Not sure
C13	Have you menstruated since giving birth?	1. Yes (go to C15)<br>2. No
C14	If not, will you continue breastfeeding once your period resume? **(then go to C17)**	1. Yes<br>2. No, why?<br>8. Already weaned<br>9. Haven't made up mind
C15	How long after delivery did your period resume?	_________months
C16	Are you still breastfeeding after your period resumed?	1. Yes<br>2. No, why?<br>8. Already weaned before period resumed
C17	Were your baby admitted to a hospital for illness treatment within one month after birth?	1. Yes<br>2. No
C18	Were your baby diagnosed with neonatal hypoglycemia?	1. Yes<br>2. No
C19	Were your baby diagnosed with neonatal jaundice?	1. Yes<br>2. No

C20	Did your baby have the runs in the past two weeks?	1. Yes<br>2. No
C21	Did your baby have respiratory diseases (e.g. cold, tracheitis, bronchitis, pneumonia, etc.) in the past two weeks?	1. Yes<br>2. No
C22	Were you or your family worried about the baby gaining too much or too little weight after birth?	1. Yes, we were worried about gaining too much weight (go to C22b)<br>2. Yes, we were worried about gaining too little weight<br>3. No (go to D1)
C22a	Worried about the baby gaining too little weight, what changes have you made in feeding approach? (then go to D1)	1. Increased breastfeeding frequency or time<br>2. Mother ate more food<br>3. Gave infant formula<br>4. Gave supplementary food<br>5. Made no changes
C22b	Worried about the baby gaining too much weight, what changes have you made in feeding approach?	1. Reduced breastfeeding frequency or time<br>2. Mother ate less food<br>3. Reduced the intake of infant formula<br>4. Made no changes

4. Family and community's support for breastfeeding

D1	Who offered postnatal care for you? (Single choice)	1. Husband<br>2. Mother<br>3. Mother-in-law<br>4. Father/Father-in-law<br>5. Postnatal caregivers<br>6. Nanny<br>7. Care center staff<br>8. Others______
D2	Who took care of your baby when you were under postnatal care? (Single choice)	1. You<br>2. Your husband<br>3. Your mother-in-law<br>4. Your mother<br>5. Your father/father-in-law<br>6. Postnatal caregivers<br>7. Nanny<br>8. Care center staff<br>9. Others______

D3	Did you not want to breastfeed when under postnatal care?	1. Never (go to D4a)<br>2. Rarely (go to D4a)<br>3. Occasionally<br>4. Often<br>5. All the time					
D3a	Why did you not want to breastfeed when under postnatal care?	1. Childbirth pain/fatigue<br>2. Nipple cracks/soreness<br>3. Low milk supply<br>4. Nipple retraction<br>5. Improper breastfeeding position<br>6. Bad mood<br>7. Frequent breastfeeding<br>8. Sleep debt<br>9. Got on a diet to regain a desired shape<br>10. Others______					
Which of the following views do people around you share?<br>1. Breast milk is more nutritious than infant formula.<br>2. Infant formula is acceptable, though not as good as breast milk.<br>3. Infant formula is as good as breast milk.<br>4. Infant formula is better than breast milk.<br>8. Not applicable.<br>9. Not sure.							
D4a	Your mother	1	2	3	4	8	9
D4b	Your mother-in-law	1	2	3	4	8	9
D4c	Your husband	1	2	3	4	8	9
D4d	Your best friend (one that influenced you the most)	1	2	3	4	8	9
D4e	Your caregivers or care center staff	1	2	3	4	8	9
D4f	Your nanny	1	2	3	4	8	9
D4g	Medical staff (one that influenced you the most)	1	2	3	4	8	9
D4h	Nursing staff	1	2	3	4	8	9
D5	How you were fed as a baby?	1. Breastfed<br>2. Infant formula/milk<br>3. Breast milk + infant formula/milk<br>4. Others<br>9. No idea					
D6	Did you receive postnatal care from professional caregivers or care center?	1. No (go to D10)<br>2. Yes, from caregivers					

		3. Yes, from care center<br>4. Yes, from both
D7	Were you given any education (leaflets or materials) that promotes breast-milk?	1. Yes<br>2. No
D8	Did you receive breastfeeding instructions from postnatal caregivers or care center?	1. Yes<br>2. No
D9	How did postnatal caregivers or care center influence your breastfeeding decisions?	1. In a great and direct way<br>2. Had certain influences<br>3. Negligible impacts
D9a	Were you advised by postnatal caregivers or care center to use infant fomula?	1. Yes<br>2. No
D10	Have you joined any mom groups (e.g. WeChat groups, App groups, offline groups)?	1. Yes<br>2. No
D10a	What is the attitude of your mom groups toward breastfeeding?	1. Exclusively support breastfeeding<br>2. Support breastfeeding<br>3. Neutral<br>4. Support infant formula<br>5. Exclusively support infant formula
D10b	How did these mom groups influence your breastfeeding decisions?	1. In a great and direct way<br>2. Had certain influences<br>3. Negligible impacts
D10c	Have you received any suggestions about using infant formula from mom groups?	1. Yes<br>2. No

5. Health care service

E1	Did you make antenatal visits to health facility during this pregnancy?	1. Yes<br>2. No (go to E7)
E2a	Did your health facility (maternal & childcare center/hospital/ township health center/community health center) run any breastfeeding education programs?	1. Yes<br>2. No (go to E7)<br>3. I don’t know (go to E7)
E2	Who were responsible for breastfeeding education at your health facility (maternal & childcare center/hospital/ township health center/community health center)? (Multiple choices)	1. Doctors<br>2. Nurses<br>3. Midwives<br>4. Breastfeeding consultants<br>5. Others

E3	Did you take part in any breastfeeding education programs at your health facility?	1. Yes<br>2. No (go to E7)
E6	Did such programs help your breastfeeding in any way?	1. Yes<br>2. No
E7	How did you give birth this time?	1. Spontaneous labor<br>2. Assisted birth (lateral episiotomy, ventouse, forceps, etc.)<br>3. C-section
E7b	Did you have a severe bleeding (> 500ml) within 24 hours after birth?	1. Yes<br>2. No<br>9. I don't know
E8	Where did you give birth?	1. Maternal & childcare center/hospital at provincial (centrally-administered municipal) or above level<br>2. Maternal & childcare center/hospital at prefectural or municipal level<br>3. Maternal & childcare center/hospital at the level of county<br>4. Maternal & childcare center/hospital at town or township level<br>5. Village clinic<br>6. Home (go to F1)<br>7. Private hospital<br>8. Others
E9	How many days did you stay in hospital after delivery?<br>("00" if less than one full day; "01" if less than two full days; etc.)	____________days in hospital<br>99. Didn't stay in hospital (go to F1)
E10	Who accompanied you while in hospital? (Single choice)	1. Nursing staff<br>2. Husband<br>3. Other family members<br>4. None
E11	Did you encounter any of the breastfeeding difficulties (nipple cracks, nipple abnormalities, breast pains, blocked ducts, mastitis, etc.) while in hospital?	1. Yes<br>2. No (go to E12)
E11a	Where did you get help when you encountered breastfeeding difficulties in hospital? (Multiple choices)	1. Mother/mother-in-law<br>2. Family members or friends<br>3. Medical professionals<br>4. Nursing staff<br>5. Lactation consultants<br>6. Yourself<br>7. Others

E11c	Have the breastfeeding difficulties you encountered during your stay in the hospital finally got resolved?	1. Yes<br>2. No
E11d	Do you think these difficulties have got in your way of breastfeeding later on?	1. Yes<br>2. No
E12	Do you think the breastfeeding information, education and instructions offered by the hospital have helped you in any way?	1. Yes<br>2. No<br>3. The hospital didn't offer any.
E13	Did the hospital recommend you any breastfeeding organizations where you can ask for help after discharge?	1. Yes, where?<br>2. No (go to E14)
E13a	Have you required any help from these organizations after discharge?	1. Yes<br>2. No, why?
E14	Has the medical service center in your community/town/township or the hospital where you gave birth paid you postpartum visits or offered remote instructions?	1. Yes<br>2. No
E16	Have you had nipple cracks, nipple abnormalities, breast pains, blocked ducts, mastitis and other symptoms after hospital discharge?	1. Yes<br>2. No (go to F1)
E17	Where did you get help when you had nipple cracks, nipple abnormalities, breast pains, blocked ducts, mastitis and other symptoms after hospital discharge? (Multiple choices)	1. Close family members<br>2. Relatives or friends<br>3. Medical professionals<br>4. Postnatal caregivers/care center staff<br>5. Lactation consultants<br>6. Infant formula marketers<br>7. Traditional medicine/massage<br>8. Yourself<br>9. Others
E17a	From which online platforms have you got help to solve these difficulties after discharge?	1. Mom & infant shopping malls<br>2. Websites and platforms of doctors and hospitals<br>3. Scientific knowledge websites, official WeChat accounts and Weibo<br>4. WeChat Moments, QQ chats<br>5. Online TV series and videos<br>6. Never asked for any help through online channels<br>7. Others
E18	Have the breastfeeding difficulties you encountered after discharge finally got resolved?	1. Yes<br>2. No
E19	Do you think these difficulties have got in your way of breastfeeding later on?	1. Yes<br>2. No

6. Workplace

F1	How was your employment before childbirth?	1. Formal employment<br>2. Informal employment (go to F18)
F2	Are you covered by the paid maternity leave policy?	1. Yes<br>2. No (go to F9)
F3	How long is the paid maternity leave at your workplace (including the maternity leave stipulated by the State and the extended leave allowed by your employer)?	_______days
F5	What percentage of your regular salary can you receive during the maternity leave (on average)?	1.______ %<br>2. Not sure
F6	Have you gone back to work now?	1. Yes<br>2. No (go to F10)
F7	How many days did you actually take for your maternity leave?	_______days
F7a	If F3≥F7 (stipulated maternity leave ≥ actual maternity leave), why?	1. Stipulated maternity leave = actual maternity leave<br>2. Returned to work early to avoid undesired payroll deductions<br>3. Returned to work early to perform required work<br>4. Returned to work early because other family members could babysit<br>5. Others
F7b	If F3<F7 (stipulated maternity leave < actual maternity leave), why?	1. Took another leave to continue breastfeeding<br>2. Took another leave to babysit<br>5. Others
F9	How did you breastfeed your baby after returning to work (then go to F11)?	1. Breastfed at workplace<br>2. Commuted between home and office to breastfeed<br>3. Hand expressed/pumped breast milk at workplace and bottle-fed back home<br>4. Only after work<br>5. The baby had been weaned

F10	How do you intent to continue to breastfeed after returning to work, if you haven't resumed work yet?	1. Breastfeed at workplace<br>2. Commute between home and office to breastfeed<br>3. Hand express/pump breast milk at workplace and bottle-feed back home<br>4. Only after work<br>5. The baby will be weaned by then
F11	How long does it usually take you to commute between workplace and home?	________minutes
F12	How about the nursing hour at your workplace?	1. None<br>2. 1 hour/workday<br>3. Others______
F13	Is there a dedicated breastfeeding corner (lactation room) at your workplace?	1. Yes (go to F15)<br>2. No
F14	Is there a private space at your workplace that allows you to breastfeed baby or pump milk?	1. Yes, I have an independent office to breastfeed baby or pump milk (go to F15).<br>2. Yes, I can pump milk in restrooms (go to F15).<br>3. Yes, there are other empty rooms (go to F15).<br>4. No.
F14a	What will you do when you need to pump milk or breastfeed?	1. Hide under the office table<br>2. Cover up to pump or breastfeed in public<br>3. Just do it in public<br>4. The baby has been weaned.<br>5. Others
F15	Is there a refrigerator to store breast milk at your workplace?	1. Yes<br>2. No
F16	Does your direct superior or boss support breastfeeding?	1. Yes<br>2. Neutral<br>3. No<br>4. I don't know
F17	How many of your colleagues have chosen to breastfeed their babies?	1. Most<br>2. Half<br>3. Few
F18	How long have you taken leave or do you intend to take leave after birth?	_______days

7. Social Environment and Culture

G1	Have you received any information that encourages breastfeeding?	1. Yes<br>2. No (go to G5)
G3a	Where have you got information about breastfeeding? (Multiple choices)	1. Medical service center or hospital where you gave birth<br>2. Traditional media (TV/radio/magazine/book）<br>3. Internet (websites/Weibo/WeChat)<br>4. SMS<br>5. Others____________________
G3c	From whom have you received information about breastfeeding? (Multiple choices)	1. Close family members<br>2. Relatives or friends<br>3. Medical professionals<br>4. Postnatal caregivers/care center staff<br>5. Lactation consultants<br>6. Infant formula marketers<br>7. Hospital nursing staff<br>8. Others
G3d	What information about breastfeeding have you received? (Multiple choices)	1. Benefits of breastfeeding<br>2. Knowledge about exclusive breastfeeding<br>3. Proper breastfeeding position<br>4. Breastfeeding timetable<br>5. How to identify sufficient milk supply<br>6. Others
G4	Do you think the information you have received is useful for your breastfeeding practices?	1. Not at all<br>2. Seldom useful<br>3. Mostly useful<br>4. Very useful<br>9. I don’t know
G5	Have you been advised to feed your baby with infant formula?	1. Yes<br>2. No (go to G8)
G7	Where have you received advice about using infant formula? (Multiple choices)	1. Medical service center or hospital where you gave birth<br>2. Traditional media (TV/radio/magazine/book）<br>3. Internet (websites/Weibo/WeChat)<br>4. SMS<br>5. Others____________________

G7a	On which online platforms have you received advice about using infant formula? (Multiple choices)	1. Mom & infant shopping malls<br>2. Websites and platforms of doctors and hospitals<br>3. Scientific knowledge websites, official WeChat accounts and Weibo<br>4. WeChat Moments, QQ chats<br>5. Online TV series and videos<br>6. Others
G8	Have you received any free samples of powder milk (for pregnant women, nursing moms, infants and babies)?	1. Yes<br>2. No (go to G10)
G9	When did you receive free samples of infant formula? (Multiple choices)	1. At _________months of pregnancy<br>2. In hospital for the delivery<br>3. After discharge from hospital<br>4. Others
G10	According to your personal experience, do you think it is necessary to have lactation rooms in public area?	1. Yes<br>2. No
G11	Do you think there have been enough lactation rooms in your living area?	1. Yes<br>2. No
G12	Have you gone out less due to the inconvenience of breastfeeding in public?	1. Yes<br>2. No
G13	Have you ever fed your baby with infant formula due to the inconvenience of breastfeeding in public?	1. Yes<br>2. No
G14	Does any of the following statements apply to you?<br>1. Yes<br>2. No	
	I feel embarrassed to breastfeed in public.<br>I feel embarrassed to breastfeed in front of female relatives and friends.<br>I feel embarrassed to breastfeed in front of male relatives and friends.	1. Yes 2. No<br>1. Yes 2. No<br>1. Yes 2. No

8. Household Income

K1	What was the total income of your household in 2016?	1. RMB□□□□□□/year A8a<br>2. Refuse to answer<br>9. I don't know
K2	What was the total expenditure of your household in 2016?	1. RMB□□□□□□/year A9a<br>2. Refuse to answer<br>9. I don't know

K3	How much did your family spend on food last month?	1. RMB□□□□□□/year A10a<br>2. Refuse to answer<br>9. I don’t know
K4	How much did your family spend on infant formula last month?	1. RMB□□□□/year<br>2. Didn’t use infant formula<br>9. Refuse to answer<br>9. I don’t know
K5	How many members are there in your family (everyone that share incomes and expenditures in your family, including the baby or babies)?	□□

Surveyed by (signature): _______________Reviewed by (signature): ______________
