# Supplementary material for: Advice to use infant formula and free samples are common in both urban and rural areas in China: a cross-sectional survey
Source: Public Health Nutr. 2021 Jan 8;24(8):1977–88. doi: 10.1017/S1368980020005364 (PMC8145468; doi:10.1017/S1368980020005364)
Supplement: Supplementary file 1 [file S1368980020005364sup.zip › S1368980020005364sup003.docx]

| Policies | Issuing organization | Released year | Currently effective | Expired year |
| --- | --- | --- | --- | --- |
| International Code of Marketing of Breast-milk Substitutes | World Health Assembly | 1981 | Yes | / |
| Maternal and Infant Health Care Law | National People’s Congress of China | 1995 | Yes | / |
| Advertising Law | National People’s Congress of China | 1995 | Yes | / |
| Administrative Measures for the Marketing of Breastmilk Substitutes | Ministry of Health, Ministry of Domestic Trade, State Administration for Industry and Commerce etc. | 1995 | No | 2017 |
| Implementation Measures of Maternal and Infant Health Care Law | State Council of the People’s Republic of China | 2001 | Yes | / |
| Decree 26 Administrative Measures for the Registration of Recipes for Formula Powder Products for Infants and Young Children | The China Food and Drug Administration | 2016 | Yes | / |
| Self-discipline Rules of Milk Powder Advertisements | China Advertising Association | 2019 | Yes | / |

**Appendix A: Selected policies for the regulation of breastmilk-substitutes marketing and promotion in China**
